# Phenotypic and metabolic features of mouse diaphragm and gastrocnemius muscles in chronic lung carcinogenesis: influence of underlying emphysema

**DOI:** 10.1186/s12967-016-1003-9

**Published:** 2016-08-23

**Authors:** Anna Salazar-Degracia, David Blanco, Mònica Vilà-Ubach, Gabriel de Biurrun, Carlos Ortiz de Solórzano, Luis M. Montuenga, Esther Barreiro

**Affiliations:** 1 Pulmonology Department-Muscle Wasting and Cachexia in Chronic Respiratory Diseases and Lung Cancer Research Group, IMIM-Hospital del Mar, Parc de Salut Mar, Health and Experimental Sciences Department (CEXS), Universitat Pompeu Fabra (UPF), Barcelona Biomedical Research Park (PRBB), C/Dr. Aiguader, 88, 08003 Barcelona, Spain; 2 Centro de Investigación en Red de Enfermedades Respiratorias (CIBERES), Instituto de Salud Carlos III (ISCIII), Barcelona, Spain; 3 Laboratorio de Biomarcadores, Programa de Tumores Sólidos, Centro de Investigación Médica Aplicada, Universidad de Navarra, Pamplona, Navarra Spain; 4 Laboratorio de Imagen del Cáncer, Programa de Tumores Sólidos, Centro de Investigación Médica Aplicada, Universidad de Navarra, Pamplona, Navarra Spain; 5 Departamento de Histología y Anatomía Patológica, Facultades de Medicina y Ciencias, Universidad de Navarra, Pamplona, Spain; 6 IDISNA, Instituto de Investigaciones Sanitarias de Navarra, Pamplona, Spain

**Keywords:** Lung carcinogenesis-induced muscle mass loss, Underlying emphysema, Respiratory and limb muscle atrophy and structural abnormalities, Muscle apoptotic nuclei, Muscle-specific proteins, PPARs proteolytic and autophagy markers

## Abstract

**Background:**

Muscle wasting negatively impacts the progress of chronic diseases such as lung cancer (LC) and emphysema, which are in turn interrelated.

**Objectives:**

We hypothesized that muscle atrophy and body weight loss may develop in an experimental mouse model of lung carcinogenesis, that the profile of alterations in muscle fiber phenotype (fiber type composition and morphometry, muscle structural alterations, and nuclear apoptosis), and in muscle metabolism are similar in both respiratory and limb muscles of the tumor-bearing mice, and that the presence of underlying emphysema may influence those events.

**Methods:**

Diaphragm and gastrocnemius muscles of mice with urethane-induced lung cancer (LC-U) with and without elastase-induced emphysema (E–U) and non-exposed controls (N = 8/group) were studied: fiber type composition, morphometry, muscle abnormalities, apoptotic nuclei (immunohistochemistry), and proteolytic and autophagy markers (immunoblotting) at 20- and 35-week exposure times. In the latter cohort, structural contractile proteins, creatine kinase (CK), peroxisome proliferator-activated receptor (PPAR) expression, oxidative stress, and inflammation were also measured. Body and muscle weights were quantified (baseline, during follow-up, and sacrifice).

**Results:**

Compared to controls, in U and E–U mice, whole body, diaphragm and gastrocnemius weights were reduced. Additionally, both in diaphragm and gastrocnemius, muscle fiber cross-sectional areas were smaller, structural abnormalities, autophagy and apoptotic nuclei were increased, while levels of actin, myosin, CK, PPARs, and antioxidants were decreased, and muscle proteolytic markers did not vary among groups.

**Conclusions:**

In this model of lung carcinogenesis with and without emphysema, reduced body weight gain and muscle atrophy were observed in respiratory and limb muscles of mice after 20- and 35-week exposure times most likely through increased nuclear apoptosis and autophagy. Underlying emphysema induced a larger reduction in the size of slow- and fast-twitch fibers in the diaphragm of U and E–U mice probably as a result of the greater inspiratory burden imposed onto this muscle.

**Electronic supplementary material:**

The online version of this article (doi:10.1186/s12967-016-1003-9) contains supplementary material, which is available to authorized users.

## Background

Skeletal muscle dysfunction and wasting are common features of patients with chronic respiratory conditions and cancer [1–4]. These frequently occurring systemic manifestations impact the patients’ exercise capacity leading to the development of early fatigue [1–5]. Cachexia, characterized by severe body and muscle mass loss and systemic inflammation, negatively impacts the quality of life of the patients and is usually present in advanced stages of their disease. In animal models of cachexia and muscle wasting of different etiology (respiratory, cardiac and cancer-induced cachexia), similar features to those observed in patients have also been reported [6–11].

Recent evidence reported by our group and others [3, 4, 6–20] has shown that several cellular and molecular mechanisms are clearly involved in the pathophysiology of muscle mass loss in patients with muscle wasting [3, 4, 16–20], as well as in different experimental models of cachexia [6–15]. The most relevant mechanisms are part of a cascade of events that lead to enhanced muscle protein breakdown [4, 16, 18], through several cellular signaling pathways [4, 16, 18], mitochondria respiratory chain dysfunction [6, 10, 14, 19, 21–23], systemic inflammation [4, 5, 24], muscle and systemic oxidative stress [4, 6, 16, 25–27], apoptosis [7], and autophagy [4, 28]. Moreover, in patients with chronic obstructive pulmonary disease (COPD) and lung cancer (LC) [4, 24], landmark structural and ultrastructural abnormalities (sarcomere disruptions) were also demonstrated in the vastus lateralis, which were associated with a significant myosin heavy chain (MyHC) loss in those muscles [4, 17]. Moreover, in COPD patients including a few with muscle mass loss, the diaphragm [17, 29, 30] has been consistently less severely affected than the lower limb muscles, in which significant signs of atrophy and proteolysis have been reported [4, 16, 18, 20, 23, 25–27, 31]. Nonetheless, we previously showed that in mice with elastase-induced emphysema, a well-validated model of chronic emphysema [9, 32–34], more prominent signs of muscle mass and MyHC loss were observed in the diaphragm than in the limb muscle.

Patients with either COPD- or LC-associated cachexia exhibited similar molecular and cellular features related to muscle mass loss in their vastus lateralis and blood [4]. In fact, the study was aimed to demonstrate that the late events taking place in the skeletal muscle of patients with cachexia of respiratory etiology (either COPD or LC) shared similar molecular and cellular profiles regardless of the underlying condition [4]. On the other hand, it has also been repeatedly demonstrated that COPD per se, especially lung emphysema, is a major risk factor for LC and that the two conditions often coexist in the same patients [35–41]. Thus, more severe muscle wasting may occur in patients with LC and underlying emphysema.

Importantly, the experimental models of cachexia in which the underlying biological mechanisms have been identified so far were mostly characterized by a subacute exposure of the animals to a large burden of different cancer cell types that led to a rapid muscle mass loss with implications in total body weight [7, 8, 14, 42]. Whether similar features in skeletal muscles are to be expected in a chronic model of lung carcinogenesis, which may mimic more closely the human disease, remains to be answered. Also, identification of whether the expression of biological events involved in muscle metabolism that maintain muscle mass may vary throughout time in wasted muscles of experimental cachexia remains an open question. Besides, the analysis of the main respiratory muscle, the diaphragm, will also be of interest in order to elucidate the potential contribution of local (lung disease) and systemic factors.

On the basis of this, we hypothesized that muscle atrophy and body weight and muscle mass loss may also develop in an experimental model of lung carcinogenesis. Furthermore, it was also hypothesized that the profile of structural and molecular events involved in protein metabolism and muscle mass maintenance are similar in both respiratory and limb muscles of mice chronically exposed to lung carcinogenesis (airways and lungs) for several relatively long periods of time in order to mimic the human condition (intermediate and late time-points). Finally, we also sought to identify whether the presence of underlying emphysema may influence the pattern of expression of the muscle biological features and mass loss in the tumor-bearing animals. Accordingly, two different experimental models were established: lung carcinogenesis with and without emphysema in mice. Hence, the study objectives were that in diaphragm and gastrocnemius of mice with urethane-induced LC with and without emphysema (elastase), the following markers were explored at two different time-points (20 and 35 weeks): (1) muscle fiber type and morphometry (sizes), (2) structural abnormalities including the number of apoptotic nuclei, (3) muscle-specific structural and functional proteins, (4) metabolic signaling factors, (5) proteolytic and autophagy markers, and (6) redox balance and inflammation. Furthermore, non-exposed control groups of mice were also used for the purpose of the investigation, and body and muscle weights were evaluated at baseline and at the end of the study period in all the groups and cohorts of mice.

## Methods

(See also the online supplementary information (Additional file 1) for detailed information on all the methodologies used in the study).

### Animal experiments

#### Experimental design

Forty-eight male A/J strain mice (2-months old, 16–23 g) were used for the purpose of the investigation, which were randomly divided into two independent time-cohorts: 20 and 35 weeks. Animals were further subdivided into the following groups in each time-cohort (N = 8/group): (1) non-exposed control mice, (2) lung carcinogenesis mice induced by urethane (U group), and (3) lung emphysema-carcinogenesis mice induced by elastase and urethane (E–U group). Lung carcinogenesis was induced as a result of a single intraperitoneal injection of 1 mg/g urethane (Urethane U2500 Sigma, St. Louis, Missouri, USA). Lung emphysema was induced through a single oropharyngeal instillation of six units per 30 g body weight of porcine pancreatic elastase (PPE, EC134GI, EPC, MI, USA) 0.15 mg/100 g elastase-high purity (EC134GI, Elastin Products Company, Owensville, Missouri, USA), following previously reported methodologies [9, 32].

#### Cancer and emphysema induction protocols

**Fig. 1:**
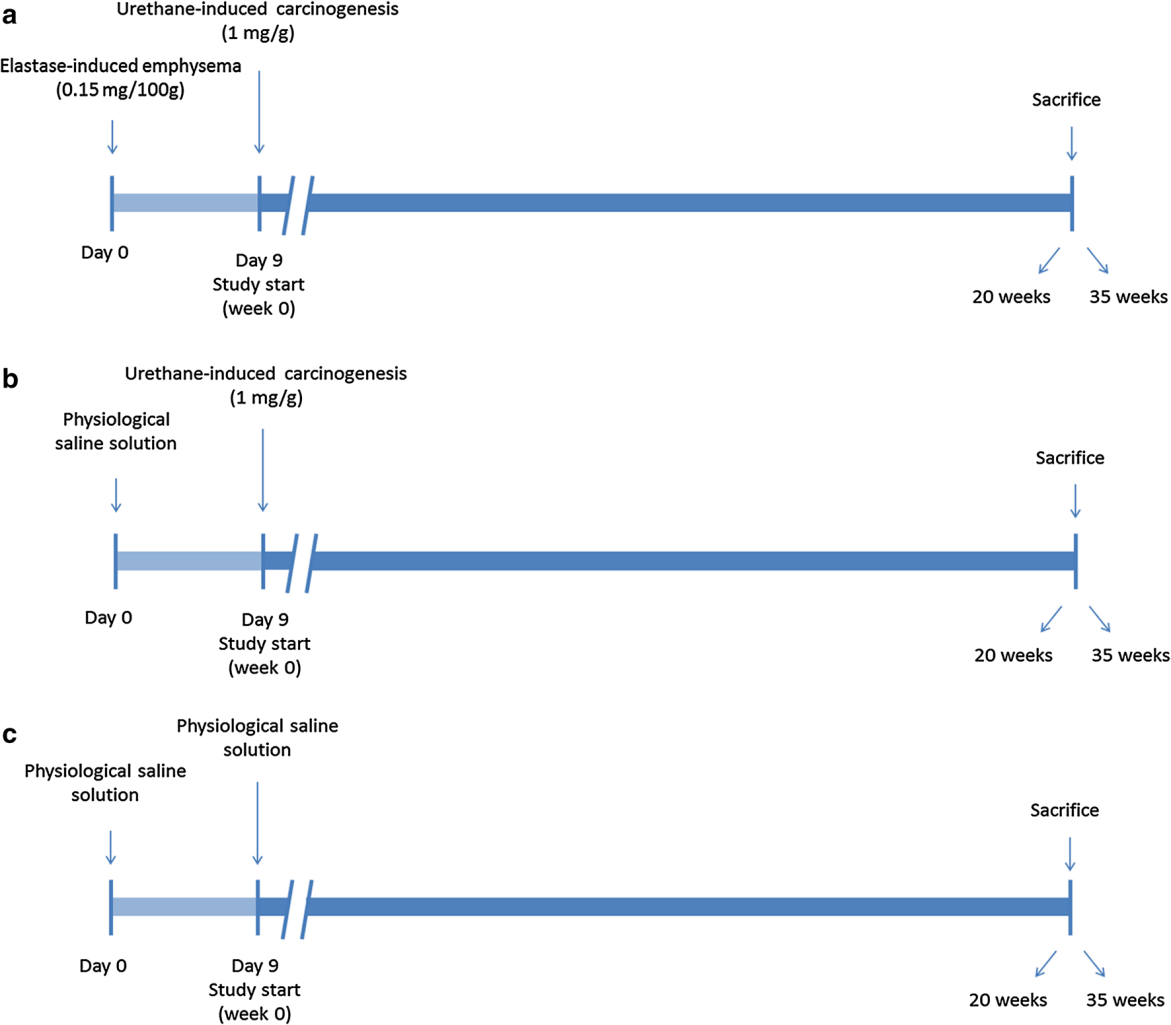
**a** Graphical time-line representation of the elastase–urethane group of mice. **b** Graphical time-line representation of the urethane group of animals. **c** Graphical time-line representation of the control group of rodents

Briefly, after administration of 2 % isoflurane in an induction box, the animals were placed on a ramp with an angle of 60°. Once immobilized, the volume of elastase was deposited in their mouth. The nostrils were occluded to force breathing through the mouth, thus enabling the mice to inhale the solution. After aspiration, the animals rested on the ramp for a few seconds to let them recover the normal breathing. Animals exposed to both emphysema and lung carcinogenesis received an oropharyngeal instillation of elastase on day 0 together with a single intraperitoneal injection of urethane on day 9 (Fig. 1a). The lung carcinogenesis group (U) received an oropharyngeal instillation of saline solution on day 0 and a single intraperitoneal injection of urethane on day 9 (Fig. 1b). Control groups were followed up for 20 or 35 weeks depending on the experimental time-cohort. These animals received oropharyngeal or intraperitoneal injections of saline solutions (0.1 mL) on days 0 or 9, respectively (Fig. 1c), as also described in previous investigations [9, 32, 34]. The different times-cohorts (20- and 35-weeks) started on day 9 after the urethane or saline (controls) injection in all the study groups.

This study was designed in accordance with the ethical regulations on animal experimentation (EU 2010/63 CEE, *Real Decreto* 53/2013 BOE 34, Spain) at the Center for Applied Medical Research (CIMA), and the Declaration of Helsinki for the use and care of animals for research purposes. Ethical approval was obtained by the Animal Experimentation Ethics Committee of CIMA in Pamplona (Spain), where the animal experiments were all conducted.

### In vivo measurements conducted on the animals

Water and food were administered to the animals for the entire duration of the study and they remained under housing environmental conditions in a 12:12 light–dark cycles. In all the mice, body weight was determined on day 0 before treatment administration and at 4- and 14-week time points, and immediately prior to their sacrifice at either 20- or 35- week time-points in each cohort [9, 32]. The percentage of body weight gain at the end of the study period was calculated as follows: [(body weight on either week 35 or 24 − body weight on day 0)]/body weight on day 0 × 100. Additionally, the percentage of muscle mass relative to final body weight in each animal was calculated as follows: [(muscle mass weight/final body weight) × 100].

#### Micro-computer tomography (CT)

Prior to sacrifice, animals underwent CT scan analyses from their lungs, which were used to verify the presence of the emphysema with and without the tumors following previously reported methodologies [32, 34]. Before the scan, animals were intraperitoneally anaesthetized prior to intubation, to be then connected to a Flexivent rodent ventilator (Scireq, Montreal, Canada). Animals were kept alive (breathing), under anesthesia (2 % isoflurane) until complete relaxation was reached. During the scan analyses, 0.5 % isoflurane was administered to the mice. All mice were scanned with an X-ray micro-CT (Micro-CAT II, Siemens Pre-Clinical Solutions, Knoxville, Tennessee, USA), with a source 80 voltage kV and a 500 microA current. Seven hundred micro-CT projections were acquired during isopressure breaths, which were hold for 650 ms (450 ms-exposure time/projection). A commercially available Dose Calculator software (Siemens Pre-clinical Solutions, Knoxville, Tennessee, USA) was used to estimate X-ray dosage-computed. In the current investigation, specific measurements of the degree of emphysema were not carried out in the animals as these analyses had been conducted in previously published studies and the model was extremely reproducible [32–34, 43].

Prior to sacrifice an intraperitoneal injection of 90 mg/kg ketamine (Imalgène^®^, Merial, Lyon, France) and 10 mg/kg xylazine (Rompun^®^, Bayer AG, Leverkusen, Germany) was administered. In all cases, the pedal and blink reflexes were evaluated in order to verify total anesthesia depth. Animals were exsanguinated to facilitate removal or organs and skeletal muscles. Immediately afterwards, in all groups of mice, the lungs, the diaphragm and gastrocnemius muscles were obtained: gastrocnemius was obtained in the first place while the animals were still breathing, subsequently to collect the diaphragm and lungs while the mice were still alive as conducted in previous studies [7–9, 32]. All muscle specimens of the 20-week cohort were immersed in buffered 4 % formaldehyde for 24 h and washed in several alcohol steps prior to paraffin-embedding. In the 35-week cohort of animals, the muscles, which were of larger size, were split in two samples, one of which was also immediately fixed in 4 % formaldehyde, rinsed in alcohols and embedded in paraffin, and the rest of the tissue was immediately frozen in liquid nitrogen to be subsequently preserved at −80 °C until further use. Paraffin-embedded tissues were used to analyze the histological features of tumors in the lungs, while in the muscles, fiber types, potential structural abnormalities, and apoptotic nuclei counts were determined. Moreover, frozen muscle specimens were used for immunoblotting and enzyme-linked immunoabsorbent assay (ELISA) procedures.

### Molecular biology analyses

All biological analyses were performed in the same laboratory at *IMIM*-*Hospital del Mar* (Barcelona, Spain) except for the lung histological analyses that were performed at CIMA (Pamplona, Spain).

#### Histological analyses of the lungs

Lung carcinogenesis histology was evaluated on 3 μm paraffin-embedded sections of the lungs in U and E–U groups of mice, according to previously published methodologies [32–34, 43]. Lobe sections were stained with hematoxylin and eosin (H&E). Images of the sections were taken at 50× and 200× under light microscopy (Zeis Axioplan 2ie microscope, Carl Zeiss, Jena, Germany).

#### Muscle fiber counts and morphometry

Diaphragm and gastrocnemius muscle fibers were identified on 3 μm thick paraffin-embedded sections in all groups of mice and the two time-cohorts. MyHC-I and MyHC-II isoforms were identified using anti-MyHC-I (clone MHC, Biogenesis Inc., Poole, England, UK) and anti-MyHC-II antibodies (clone MY-32, Sigma, Saint Louis, MO), respectively, according to previously published methodologies [4, 6–8, 18].

#### Muscle structural abnormalities

The area fraction of normal and abnormal muscle was evaluated on 3 μm paraffin-embedded sections of the diaphragm and gastrocnemius muscles in all groups of mice following previously published methodologies [4, 6–8, 16].

#### Terminal deoxynucleotidyl transferase- mediated uridine 5′-triphosphate (UTP) nick- end labelling (TUNEL) assay

In muscle paraffin-embedded sections, apoptotic nuclei were identified using the TUNEL assay (In Situ Cell Death Detection Kit, POD, Roche Applied Science, Mannheim, Germany) in both diaphragm and gastrocnemius muscle specimens from all study groups following the manufacturer’s instructions and previous studies [6–8, 44]. In each muscle preparation, altered fibers were expressed as the ratio of total TUNEL positively-stained nuclei to the total number of counted nuclei, as also previously described [6–8, 44]. A minimum amount of 300 nuclei were counted in each muscle preparation. Final results corresponded to the mean value of the counts provided by the two independent observers (concordance rate 95 %).

#### Immunoblotting of 1D electrophoresis

Protein levels of the different molecular markers explored in the study muscles were analyzed in the diaphragm and gastrocnemius of the experimental groups belonging to the 35-week cohort using previously published methodologies [6–8, 44]. Protein content of the different markers was identified using specific primary antibodies: actin (anti-alpha-sarcomeric actin antibody, clone 5C5, Sigma Sigma-Aldrich, St. Louis, MO, USA), myosin heavy chain (anti-MyHC antibody, clone A4.1025, Upstate-Millipore, Temecula, CA, USA), creatine kinase (anti-creatine kinase antibody, Santa Cruz), carbonic anhydrase-3 (anti-carbonic anhydrase-3 antibody, Santa Cruz Biotechnology, Santa Cruz, CA, USA), myogenin (anti-myogenin antibody, Santa Cruz), catalase (anti-catalase antibody, Calbiochem, Darmstadt, Germany), superoxide dismutase (SOD)2 (anti-SOD2 antibody, Santa Cruz), SOD1 (anti-SOD1 antibody, Santa Cruz), malondialdehyde (MDA) protein adducts (anti-MDA protein adducts antibody Academy Bio-Medical Company, Inc., Houston, TX, USA), peroxisome proliferator-activated receptor (PPAR)-alpha (anti-PPAR-alpha antibody (H-98), Santa Cruz), PPAR-gamma (anti- PPAR-gamma antibody (H-100), Santa Cruz), PPAR gamma coactivator (PGC) -1α (anti-PGC-1 alpha antibody (H-300), Santa Cruz), total protein ubiquitination (anti-protein ubiquitination antibody, Boston Biochem, Cambridge, MA, USA), ubiquitin-conjugating enzyme E2_14k_ (anti-E2_14K_ antibody, Boston Biochem), muscle ring finger (MURF)-1 (anti-MURF-1 antibody, Santa Cruz Biotechnology, Santa Cruz, CA, USA), calpain-1 (anti-calpain-1 antibody, Cell Signaling, USA), nucleoporin p-62 (anti-p62/SQSTM1 antibody, Sigma-Aldrich, St. Louis, MO, USA), beclin-1 (anti-beclin-1 antibody, Santa Cruz), light chain (LC)3B (anti-LC3B antibody, Cell Signaling, Boston, MA, USA), and glyceraldehyde-3-phosphate dehydrogenase (GAPDH, anti-GAPDH antibody, Santa Cruz). Antigens from all the samples were detected using horseradish peroxidase (HRP)-conjugated secondary antibodies (Jackson ImmunoResearch Inc, West Grove, PA, USA) and a chemiluminescence kit (Thermo Scientific, Rockford, IL, USA).

#### Cytokine ELISA

Protein levels of the inflammatory cytokines tumor necrosis factor (TNF)-α and interleukin (IL)-6 were quantified in the diaphragm and gastrocnemius muscles in all study groups of the 35-week cohort using specific sandwich ELISA kits (eBioscience, Bender MedSystems GmbH, Vienna, Austria), and specific manufacturer’s instructions and previous studies were followed [4, 6, 25, 26].

### Statistical analysis

The normality of the study variables was verified using the Shapiro–Wilk test. Physiological and biological results are represented as mean (standard deviation) and the comparisons between all study groups were analyzed using the one-way analysis of variance (ANOVA), in which *Tukey* post hoc analysis was used to adjust for multiple comparisons among the three study groups in each time-cohort (20 and 35 weeks) independently. For the purpose of the investigation, results obtained in the animals and those obtained in the study muscles were subsequently analyzed as follows: (1) non-exposed controls versus lung carcinogenesis mice (U group), (2) non-exposed controls versus lung emphysema-carcinogenesis mice (E–U group), and (3) U versus E–U groups of animals. The sample size chosen was based on previous studies [6–9, 11, 14, 45, 46] and on assumptions of 80 % power to detect an improvement of more than 20 % in measured outcomes at a level of significance of p ≤ 0.05. In most biological variables, mean difference between groups was initially estimated at a minimum of 20–25 % and standard deviation was approximately 25–30 % of the mean value for each of the variables. All statistical analyses were performed using the Statistical Package for the Social Sciences (Portable SPSS, PASW statistics 12.0 version for windows, SPSS Inc., Chicago, IL, USA).

## Results

### Physiological characteristics

**Fig. 2:**
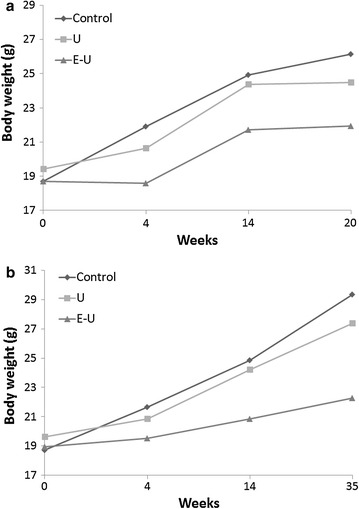
**a** Graphical representation of the progression of body weight in mice from all experimental groups over the study period in the 20-week time-cohort. The following signs have been used in each group: control (*diamonds*), U group (*squares*), and E–U group (*triangles*). **b** Graphical representation of the progression of body weight in mice from all experimental groups over the study period in the 35-week time-cohort. The following signs have been used in each group: control (*diamonds*), U group (*squares*), and E–U group (*triangles*). *E–U* elastase–urethane, *U* urethane

As shown in Table 1 and Fig. 2, body weight at the end of study period (20- and 35-time-points) was significantly reduced in E–U mice compared to non-exposed controls in both time-cohorts. Nonetheless, body weight gain was significantly reduced in both U and E–U groups compared to the non-exposed controls in each time-cohort (Table 1). Diaphragm and gastrocnemius muscle weights were significantly reduced in U and E–U groups compared to non-exposed control animals in both 20-week and 35-week cohorts (Table 1). However, compared to control animals, the percentage of diaphragm weight to total body weight significantly decreased only in U mice of both time-cohorts, whereas in the gastrocnemius, a significant reduction in this parameter was observed only in U mice of the 35-week cohort (Table 1).

**Table 1:** Physiological characteristics of all study groups of mice

	Controls	U	E–U
20 weeks			
Initial body weight (g)	18.7 (0.67)	19.4 (1.91)	18.7 (1.55)
Final body weight (g)	26.2 (1.38)	24.5 (2.18)	21.9 (1.50)***,^§^
Body weight gain (%)	+39.8 (6.58)	+26.3 (5.46)*	+17.8 (10.8)***
Diaphragm weight (g)	0.08 (0.01)	0.06 (0.004)***	0.06 (0.01)***
Diaphragm (% body weight)	0.30 (0.05)	0.25 (0.02)*	0.26 (0.04)
Gastrocnemius weight (g)	0.15 (0.004)	0.12 (0.01)***	0.12 (0.01)***
Gastrocnemius (% body weight)	0.56 (0.04)	0.50 (0.04)	0.55 (0.05)
35 weeks			
Initial body weight (g)	18.7 (0.67)	19.6 (1.61)	18.9 (2.71)
Final body weight (g)	29.4 (2.22)	27.4 (1.41)	22.3 (2.54)***^, §§^
Body weight gain (%)	+56.9 (10.1)	+39.9 (8.40)*	+18.1 (5.45)***^, §^
Diaphragm weight (g)	0.09 (0.02)	0.06 (0.01)**	0.06 (0.01)**
Diaphragm (% body weight)	0.30 (0.04)	0.22 (0.04)*	0.28 (0.05)
Gastrocnemius weight (g)	0.15 (0.01)	0.10 (0.02)***	0.10 (0.01)***
Gastrocnemius (% body weight)	0.50 (0.03)	0.37 (0.07)**	0.47 (0.07)^§^

Variables are presented as mean (standard deviation)

*U* urethane, *E–U* elastase–urethane, *g* gram

Statistical significance: * p ≤ 0.05, ** p ≤ 0.01 and *** p ≤ 0.001 between any of the experimental groups and the control mice

^§^p ≤ 0.05 and ^§§^p ≤ 0.01 between urethane and elastase–urethane groups of mice

### Micro-CT imaging results

**Fig. 3:**
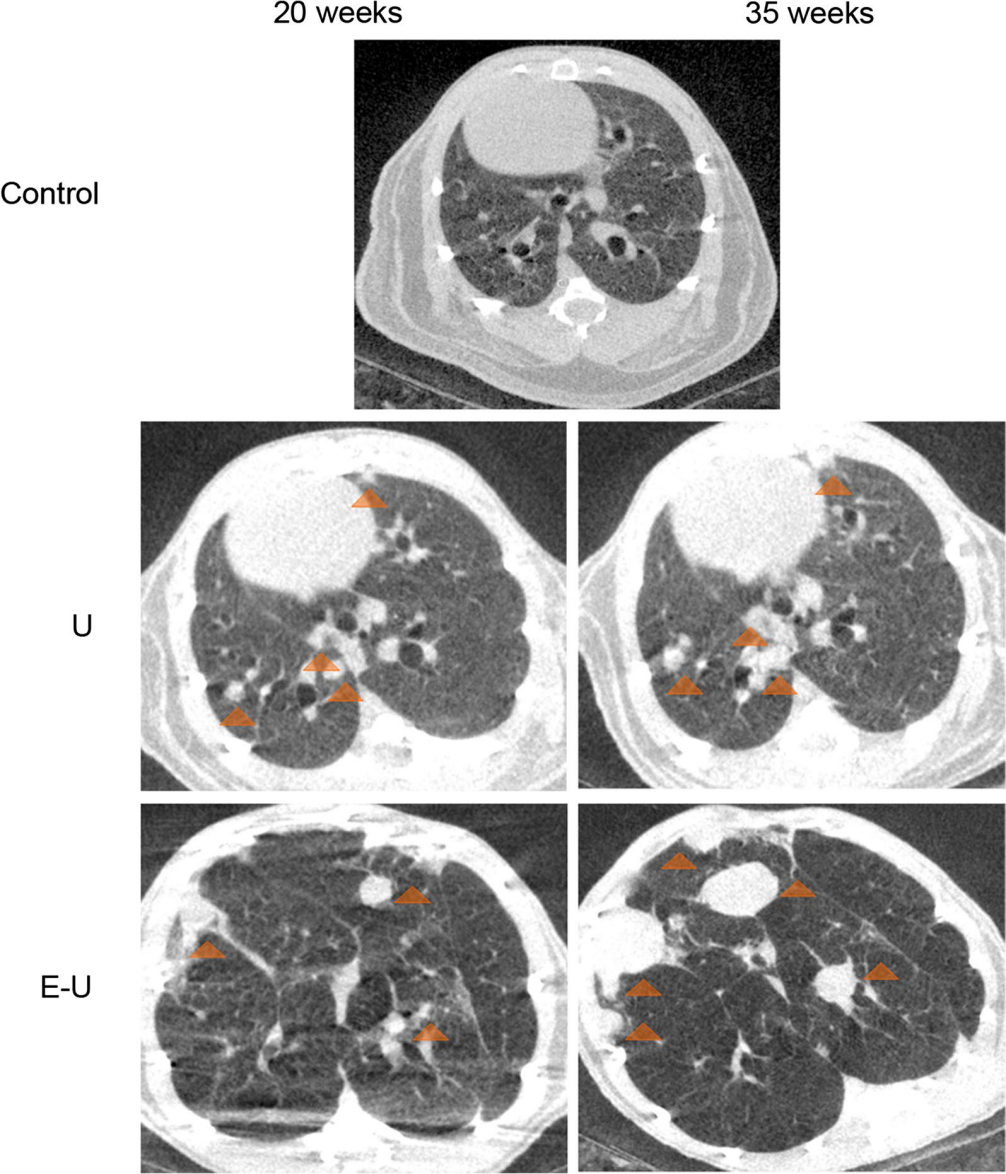
Representative images of micro-CT scan analyses in all study groups in the 20- (*left panel*) and 35-week (*right panel*) cohorts of mice. Lung nodules (*orange triangles*) are shown in micro-CT slices of lung parenchyma for U (*middle panel*) and E–U (*bottom panel*) groups of mice. *E–U* elastase–urethane, *U* urethane

The results obtained from the micro-CT analyses of the longitudinal subgroups confirm the feasibility of conducting in vivo longitudinal studies of these models in both 20- and 35-week time-cohorts. In both U and E–U groups of mice, the appearance of the nodules, which were identified as hyper intense convex masses (most of the solid structures seen in the CT-images), were found well-circumscribed, in juxta pleural or juxta vascular areas of the lungs (Fig. 3). In both time-cohorts, the intensity of the lung parenchyma was lower in E–U (Fig. 3, bottom panels) animals compared to U mice (Fig. 3, middle panels) as a result of the lung tissue loss induced by elastase, while lung volume distension was observed in the same animals (Fig. 3).

### Lung histological characteristics

**Fig. 4:**
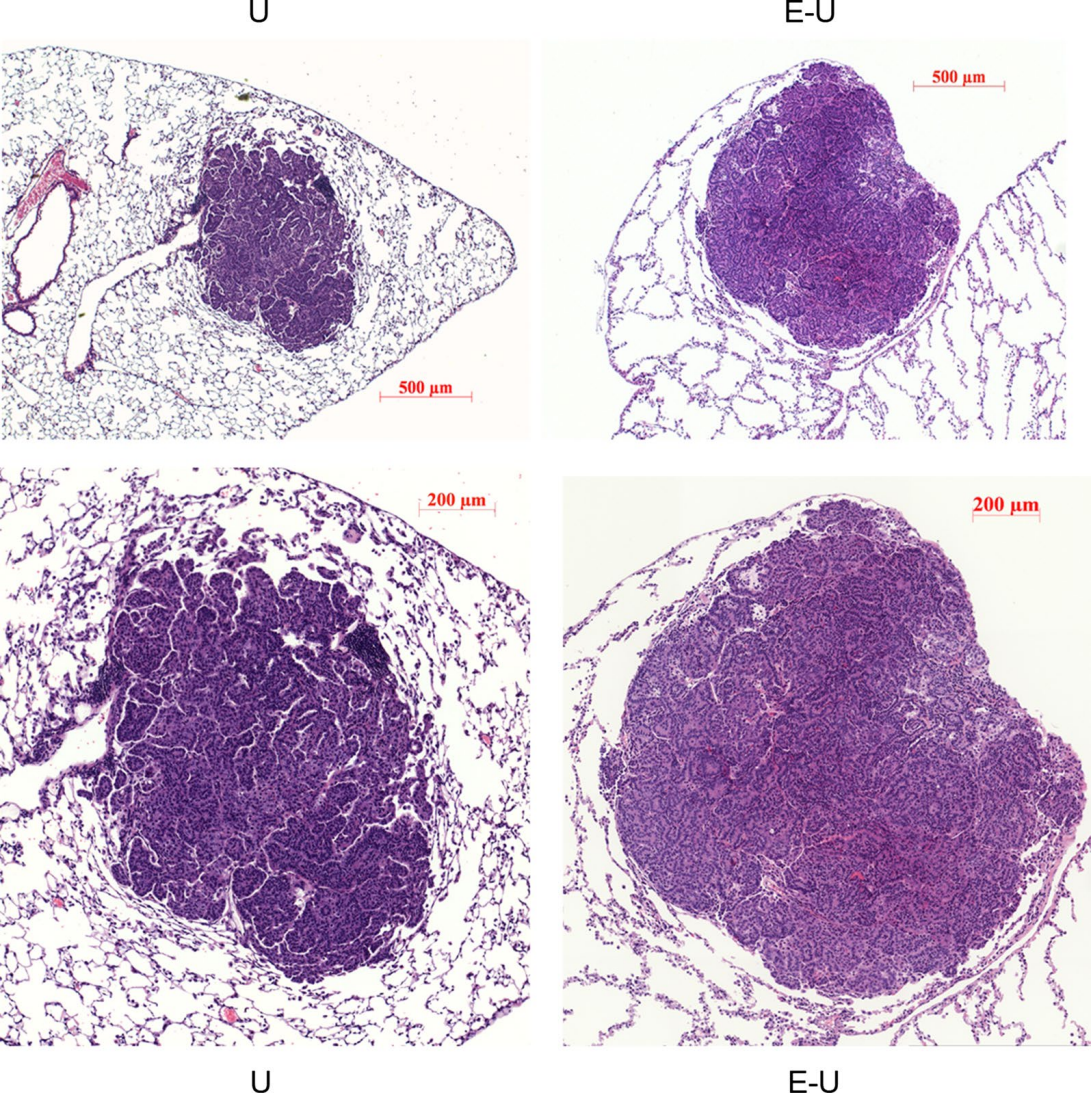
Representative examples of histological sections stained with hematoxylin and eosin (×5 and ×20) of whole individual tumors in U (*left panels*) and E–U (*right panels*) groups of mice. *E–U* elastase–urethane, *U* urethane

In accordance with the radiological data, histological features confirmed the presence of emphysema in all the mice that had been treated with elastase (E–U group) of both time-cohorts. Namely, the alveolar walls were remarkably thin and the alveolar spaces were enlarged (Fig. 4). The tumor lesions observed in the U mice (Fig. 4, left panels) were histologically similar to those observed in the E–U animals (Fig. 4, right panels), with the exception of the surrounding parenchyma, which was relatively normal in the U animals (non-emphysematous lungs, Fig. 4, left panels). The lesions were mostly adenomas or adenomatous proliferative lesions, a few of them in close or direct contact with club cells lining the bronchioles. The largest lesions in both groups and cohorts displayed the classical adenocarcinoma phenotype, as shown by presence of nuclear atypia, papillary morphology, and invasive margins (Fig. 4).

### Muscle structural characteristics

#### Fiber type composition

**Fig. 5:**
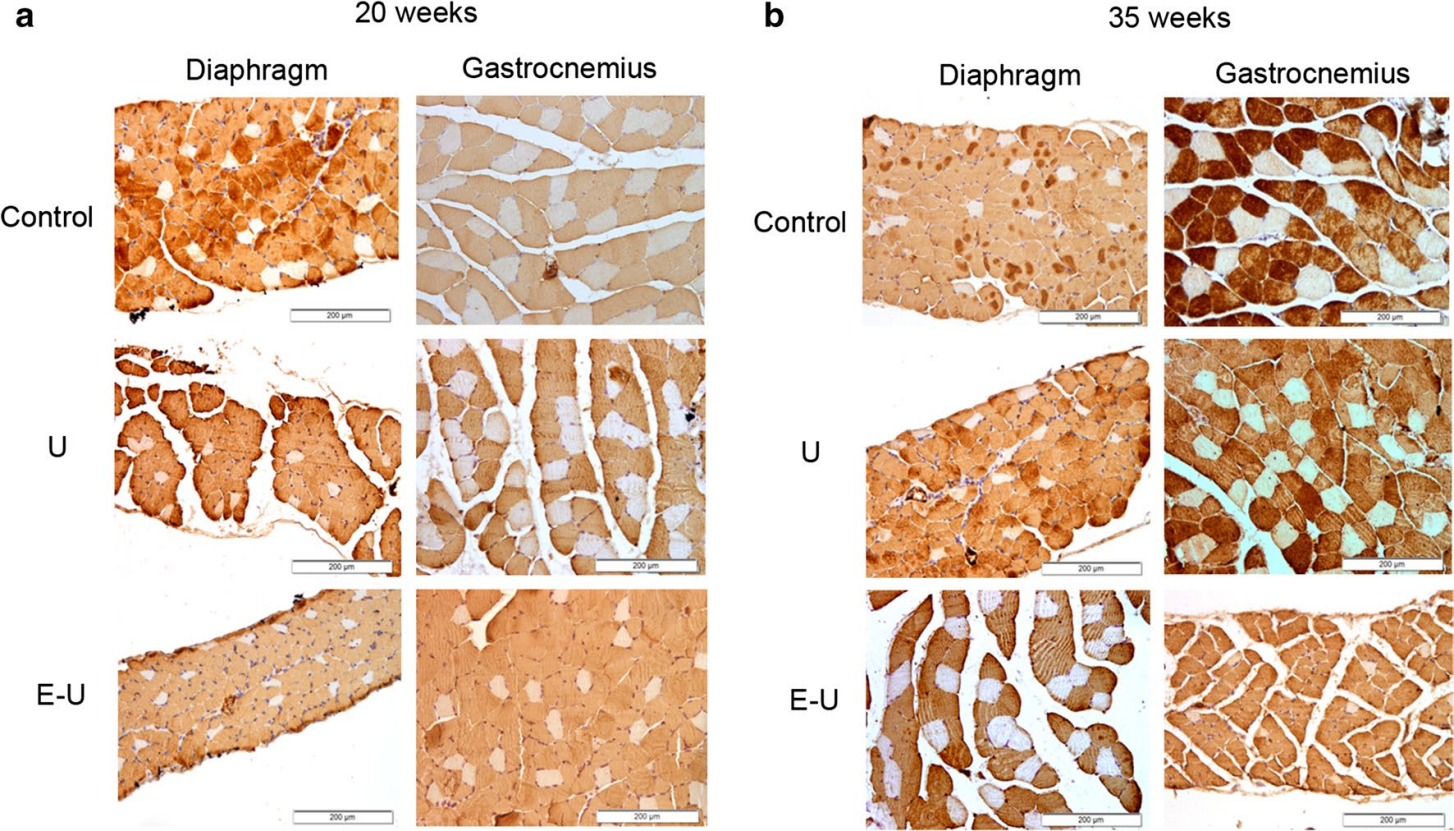
**a** Representative examples of stained muscle fibers in diaphragm and gastrocnemius muscles of control (*top panel*), U group (*middle panel*) and E–U group (*bottom panel*) in the 20-week cohort of animals. Myofibers positively stained with the anti-MyHC type II antibody appear in brown color (*calibration bar* 200 μm). Type I fibers appear in white color (not stained). **b** Representative examples of stained muscle fibers in diaphragm and gastrocnemius muscle of control (*top panel*) and U group (*middle panel*) and E–U group (*bottom panel*) in the 35-week cohort of animals. Myofibers positively stained with the anti-MyHC type II antibody appear in brown color (*calibration bar* 200 μm). Type I fibers appear in white color (not stained). *anti-MyHC* anti-myosin heavy chain, *E–U* elastase–urethane, *U* urethane

**Fig. 6:**
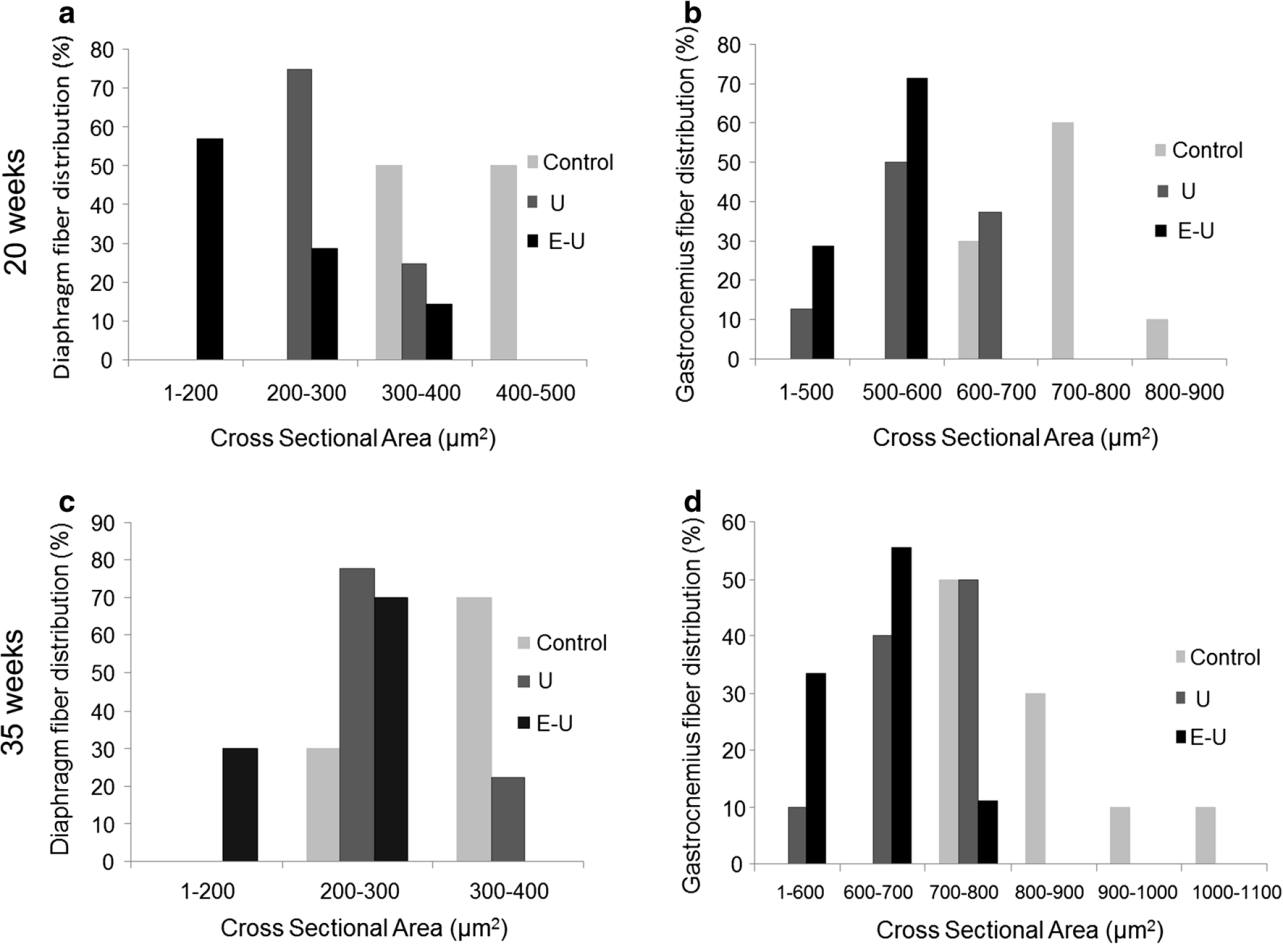
Schematic representation of the distribution of the cross-sectional areas of the muscle fibers in the diaphragm and gastrocnemius muscles in both 20- and 35-week time cohorts of animals (**a**–**d**, respectively). Values of cross-sectional areas in the control mice are represented in *light grey bars*, while those detected in U and E–U animals are represented in *dark grey* and *black* respectively in all the histograms (**a**–**d**)

In the 20- and 35-week cohorts of mice, proportions of type I and type II fibers did not significantly differ in any muscle among the experimental groups (Tables 2, 3; Fig. 5a, b). As expected, muscle fiber cross-sectional areas were significantly decreased in the diaphragm and gastrocnemius muscles of U and E–U mice in both 20- and 35-week time cohorts compared to control animals (Tables 2, 3; Fig. 5a, b). Atrophy was found in both slow- and fast-twitch fibers in both types of muscles. Additionally, in the diaphragm of E–U mice, the size of type I and type II fibers was significantly reduced when compared to U animals in both 20- and 35-week time cohorts (Tables 2, 3; Fig. 5a, b). Furthermore, the distributions of the muscle fiber cross-sectional areas in respiratory and limb muscles of all study groups in both 20- and 35-week time-cohorts are depicted in Fig. 6.

**Table 2:** Muscle structure in both diaphragm and gastrocnemius muscles of mice of the 20-week cohort

	Controls	U	E–U
Diaphragms			
Fiber type proportions			
Type I fibers (%)	8.9 (1.66)	9.5 (1.70)	8.3 (1.05)
Type II fibers (%)	91.1 (1.66)	90.5 (1.70)	91.7 (1.05)
Cross-sectional area			
Type I fibers (μm^2^)	342.7 (61.9)	235.7 (32.5)***	155.3 (41.5)***^, §^
Type II fibers (μm^2^)	473.2 (43.0)	328.2 (30.4)***	230.6 (50.5)***^, §§§^
Gastrocnemius			
Fiber type proportions			
Type I fibers (%)	25.1 (7.28)	20.6 (4.82)	20.7 (6.74)
Type II fibers (%)	74.9 (7.28)	79.4 (4.82)	79.3 (6.74)
Cross-sectional area			
Type I fibers, (μm^2^)	746.6 (69.6)	504.5 (123.5)***	491.4 (47.3)***
Type II fibers, (μm^2^)	691.4 (65.7)	622.4 (61.8)*	568.5 (31.3)***

Variables are presented as mean (standard deviation)

*U* urethane, *E–U* elastase–urethane

Statistical significance: * p ≤ 0.05 and *** p ≤ 0.001 between any of the experimental groups and the control mice

^§^p ≤ 0.05 and ^§§§^p ≤ 0.001 between urethane and elastase–urethane groups of mice

**Table 3:** Muscle structure in both diaphragm and gastrocnemius muscles of all groups of mice of the 35-week cohort

	Controls	U	E–U
Diaphragms			
Fiber type proportions			
Type I fibres (%)	8.1 (1.00)	8.2 (0.80)	7.7 (1.05)
Type II fibres (%)	91.9 (1.00)	91.8 (0.80)	92.3 (1.05)
Cross-sectional area			
Type I fibres (μm^2^)	257.9 (33.1)	221.7 (30.3)*	182.4 (12.8)***^, §^
Type II fibres (μm^2^)	377.9 (30.1)	326.7 (47.6)*	258.8 (28.1)***^, §§^
Gastrocnemius			
Fiber type proportions			
Type I fibres (%)	22.7 (6.26)	24.0 (4.73)	20.5 (3.25)
Type II fibres (%)	77.3 (6.26)	76.0 (4.73)	77.5 (3.25)
Cross-sectional area			
Type I fibres (μm^2^)	824.7 (155.2)	680.5 (67.6)*	609.8 (74.6)***
Type II fibres (μm^2^)	835.3 (100.7)	721.9 (70.4)*	665.2 (80.0)***

Variables are presented mean (standard deviation)

*U* urethane, *E–U* elastase–urethane

Statistical significance: * p ≤ 0.05 and *** p ≤ 0.001 between any of the experimental groups and the control mice

^§^p ≤ 0.05 and ^§§^p ≤ 0.01 between urethane and elastase-urethane groups of mice

#### Muscle abnormalities

**Fig. 7:**
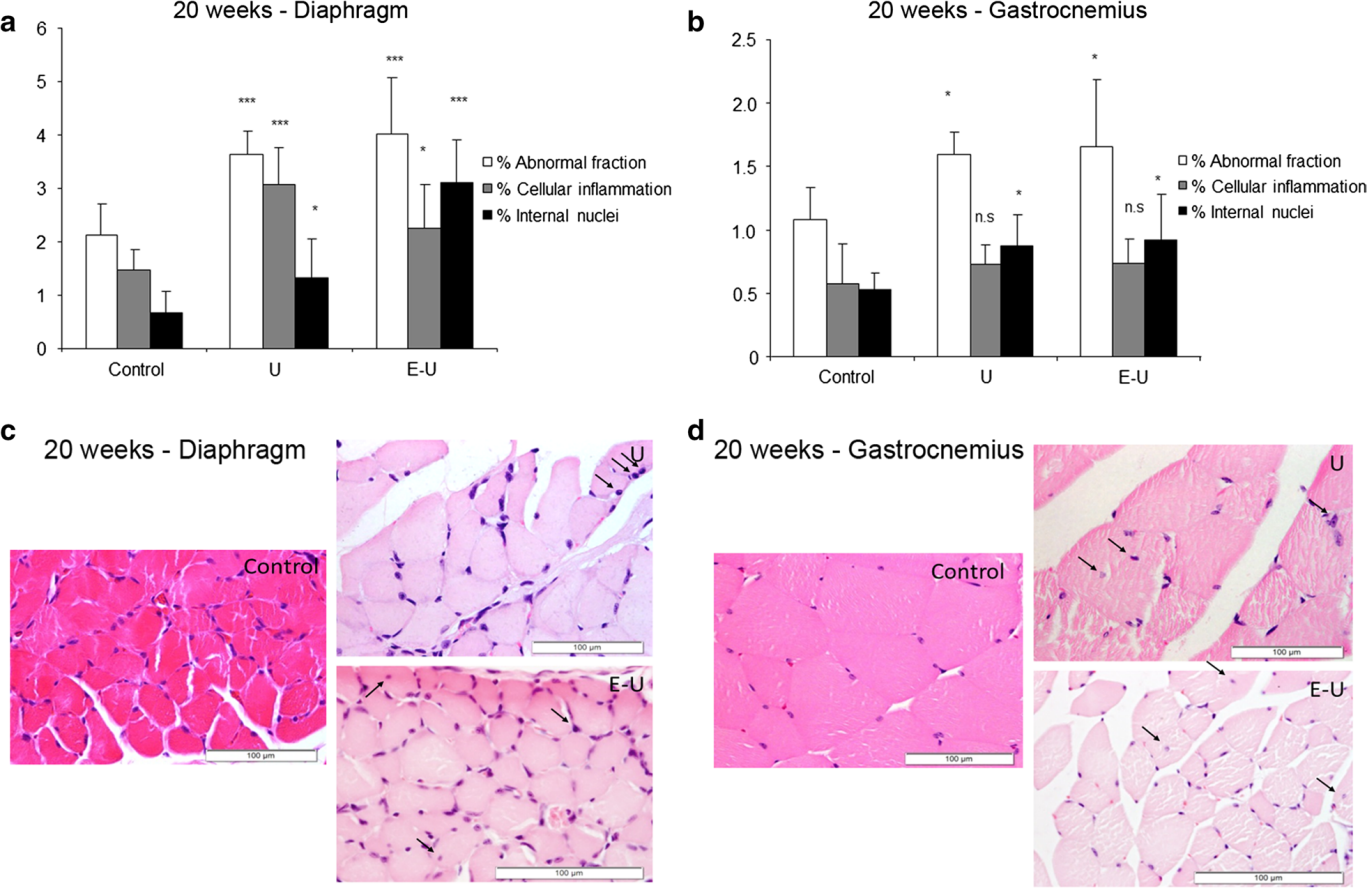
**a** Mean values and standard deviation of the percentage of abnormal fraction (*white bars*), cellular inflammation (*grey bars*) and internal nuclei (*black bars*) in the diaphragm muscle of the 20-week cohort animals. Statistical significance is represented as follows: *p ≤ 0.05 and ***p ≤ 0.001 between any of the intervention groups (U and E–U) and control mice. **b** Mean values and standard deviation of the percentage of abnormal fraction (*white bars*), cellular inflammation (*grey bars*) and internal nuclei (*black bars*) in the gastrocnemius muscle of 20-week cohort animals. Statistical significance is represented as follows: n.s.: non-significant and *p ≤ 0.05 between any of the intervention groups (U and E–U) and control mice. **c** Representative examples of muscle structural abnormalities in diaphragm of 20-week cohort animals (*calibration bar* 100 μm). A representative image of muscles in U group (*right top image*), in which *black arrows* point towards inflammatory cells, and a representative image of muscles in E–U group (*right bottom image*), in which *black arrows* point towards inflammatory cells and internal nuclei. **d** Representative examples of muscle structural abnormalities in gastrocnemius of 20-week cohort animals (*calibration bar* 100 μm). A representative image of muscles in U group (*right top image*), in which *black arrows* point towards inflammatory cells, and a representative image of E–U group (*right bottom image*), in which *black arrows* point towards internal nuclei. *E–U* elastase–urethane, *U* urethane

**Fig. 8:**
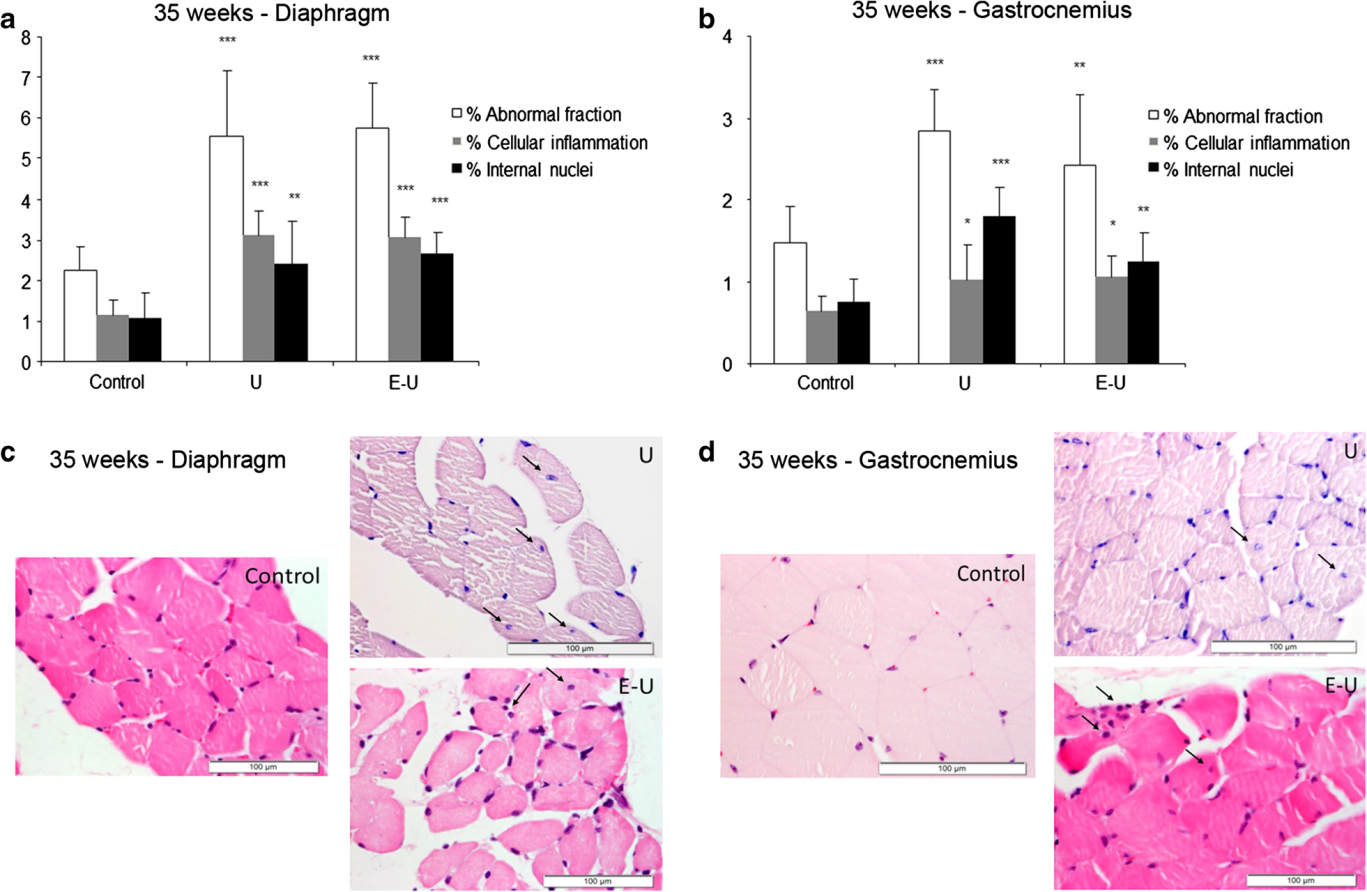
**a** Mean values and standard deviation of the percentage of abnormal fraction (*white bars*), cellular inflammation (*grey bars*) and internal nuclei (*black bars*) in the diaphragm muscle of 35-week cohort animals. Statistical significance is represented as follows: **p ≤ 0.01 and ***p ≤ 0.001 between any of the intervention groups (U and E–U) and control mice. **b** Mean values and standard deviation of the percentage of abnormal fraction (*white bars*), cellular inflammation (*grey bars*) and internal nuclei (*black bars*) in the gastrocnemius muscle of 35-week cohort. Statistical significance is represented as follows: *p ≤ 0.05, **p ≤ 0.01 and ***p ≤ 0.001 between any of the intervention groups (U and E–U) and control mice. **c** Representative examples of muscle structural abnormalities in diaphragm of 35-week cohort animals (*calibration bar* 100 μm). A representative image of muscles in U group (*right top image*), in which *black arrows* point towards internal nuclei, and a representative image of E–U group (*right bottom image*), in which *black arrows* point towards inflammatory cells and internal nuclei. **d** Representative examples of muscle structural abnormalities in gastrocnemius of 35-week cohort animals (*calibration bar* 100 μm). A representative image of muscles in U group (*right top image*), in which *black arrows* point towards internal nuclei and a representative image of E–U group (*right bottom image*), in which *black arrows* point towards inflammatory cells and internal nuclei. *E–U* elastase–urethane, *U* urethane

In the 20 week-cohort of mice, a significant increase in the proportion of muscle structural abnormalities and internal nuclei counts were found in both diaphragm and gastrocnemius muscles of U and E–U mice compared to non-exposed control animals (Fig. 7a–d). In the same cohort, the proportion of inflammatory cell counts was only significantly increased in the diaphragm muscle of U and E–U mice compared to the non-exposed controls (Fig. 7a–d). Interestingly, in the 35-week cohort, proportions of total muscle abnormalities, inflammatory cell and internal nuclei counts were significantly higher in both muscles of U and E–U mice compared to the non-exposed controls (Fig. 8a–d).

#### Muscle fiber apoptosis

**Fig. 9:**
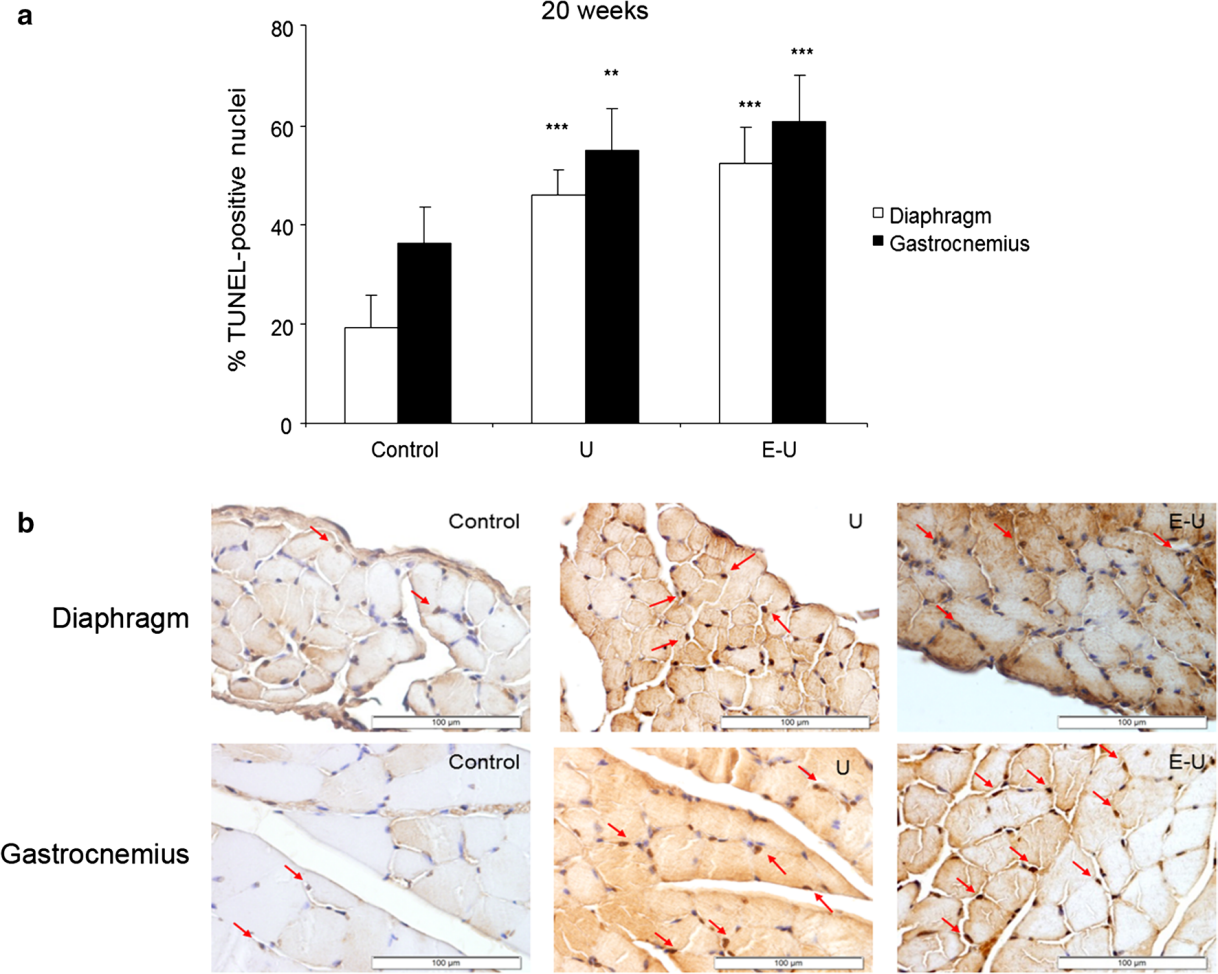
**a** Mean values and standard deviation of the percentage of positively stained nuclei for the TUNEL assay in diaphragm (*white bars*) and gastrocnemius (*black bars*) muscles of the 20-week cohort of animals. Statistical significance is represented as follows: **p ≤ 0.01 and ***p ≤ 0.001 between any of the intervention groups (U and E–U) and control mice. **b** Representative examples of positively nuclei (*red arrows*) stained for the TUNEL assay in the diaphragm (*top panel*) and gastrocnemius (*bottom panel*) muscles of the 20-week cohort of the different study groups of mice (*calibration bar* 100 μm)

**Fig. 10:**
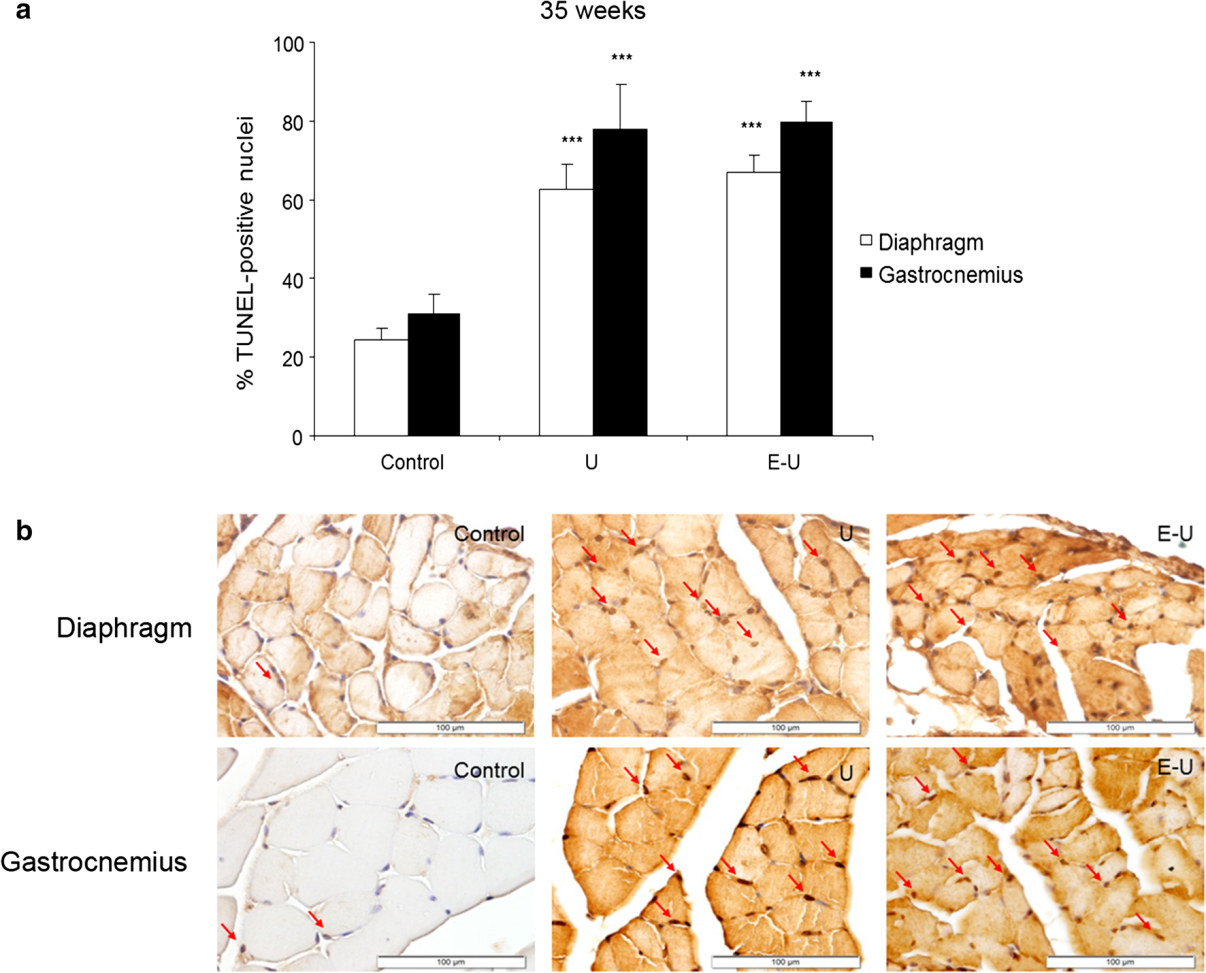
**a** Mean values and standard deviation of the percentage of positively stained nuclei for the TUNEL assay in diaphragm (*white bars*) and gastrocnemius (*black bars*) muscles of the 35-week cohort of animals. Statistical significance is represented as follows: ***p ≤ 0.001 between any of the intervention groups (U and E–U) and control mice. **b** Representative examples of positively nuclei (*red arrows*) stained for the TUNEL assay in the diaphragm (*top panel*) and gastrocnemius (*bottom panel*) muscles of the 35-week cohort of the different study groups of mice (*calibration bar* 100 μm). *E–U* elastase–urethane, *U* urethane

A significant rise in TUNEL positively-stained nuclei was observed in 20- and 35-week cohorts of mice in both diaphragm and gastrocnemius muscles of U and E–U mice compared to non-exposed controls (Figs. 9a, b, 10a, b, respectively).

### Contractile and functional muscle proteins

**Fig. 11:**
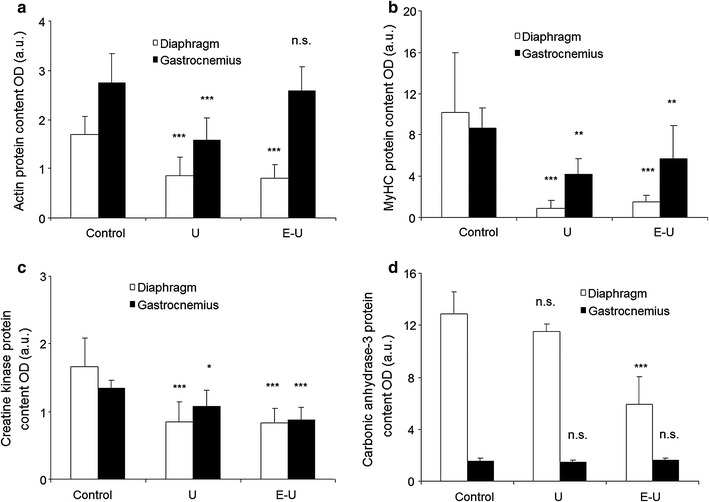
**a** Mean values and standard deviation of skeletal muscle actin, in diaphragm (*white bars*) and gastrocnemius (*black bars*) muscles as measured by optical densities. Statistical significance is represented as follows: n.s.: non-significant and ***p ≤ 0.001 between any of the intervention groups (U and E–U) and control mice. **b** Mean values and standard deviation of MyHC, in diaphragm (*white bars*) and gastrocnemius (*black bars*) muscles as measured by optical densities. Statistical significance is represented as follows: n.s.: non-significant, **p ≤ 0.01, and ***p ≤ 0.001 between any of the intervention groups (U and E–U) and control mice. **c** Mean values and standard deviation of creatine kinase (CK), in diaphragm (*white bars*) and gastrocnemius (*black bars*) muscles as measured by optical densities. Statistical significance is represented as follows: *p ≤ 0.05 and ***p ≤ 0.001 between any of the intervention groups (U and E–U) and control mice. **d** Mean values and standard deviation of carbonic anhydrase-3, in diaphragm (*white bars*) and gastrocnemius (*black bars*) muscles as measured by optical densities. Statistical significance is represented as follows: n.s.: non-significant and ***p ≤ 0.001 between any of the intervention groups (U and E–U) and control mice. *a.u.* arbitrary units, *E–U* elastase–urethane, *MyHC* myosin heavy chain, *OD* optical densities, *U* urethane

All the analyses of contractile and functional muscle proteins were carried out in the 35-week cohort mice. Protein levels of skeletal muscle actin were significantly reduced in both diaphragm and gastrocnemius of U mice compared to the non-exposed controls, while in the E–U group, a significant decline in actin levels was only seen in the diaphragm (Fig. 11a; Additional file 1: Figure S3). Protein levels of MyHC and creatine kinase decreased in both diaphragm and gastrocnemius of U and E–U mice compared to the non-exposed controls (Fig. 11b, c; Additional file 1: Figures S4, S5). In E–U mice, carbonic anhydrase-3 levels were only significantly reduced in the diaphragm compared to the non-exposed controls (Fig. 11d; Additional file 1: Figure S6).

### Muscle growth and metabolism

**Fig. 12:**
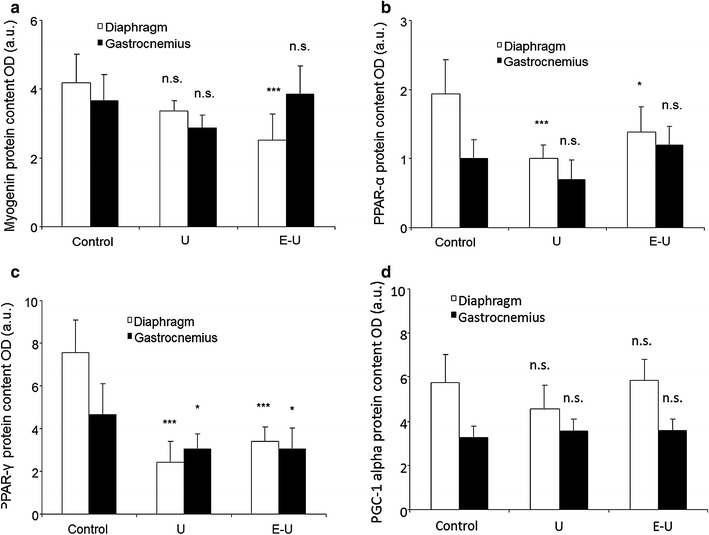
**a** Mean values and standard deviation of myogenin, in diaphragm (*white bars*) and gastrocnemius (*black bars*) muscles as measured by optical densities. Statistical significance is represented as follows: n.s.: non-significant and ***p ≤ 0.001 between any of the intervention groups (U and E–U) and control mice. **b** Mean values and standard deviation of PPAR-α, in diaphragm (*white bars*) and gastrocnemius (*black bars*) muscles as measured by optical densities. Statistical significance is represented as follows: n.s.: non-significant, *p ≤ 0.05 and ***p ≤ 0.001 between any of the intervention groups (U and E–U) and control mice. **c** Mean values and standard deviation of PPAR-gamma, in diaphragm (*white bars*) and gastrocnemius (*black bars*) muscles as measured by optical densities. Statistical significance is represented as follows: *p ≤ 0.05 and ***p ≤ 0.001 between any of the intervention groups (U and E–U) and control mice. **d** Mean values and standard deviation of PGC-1α, in diaphragm (*white bars*) and gastrocnemius (*black bars*) muscles as measured by optical densities. Statistical significance is represented as follows: n.s.: non-significant between any of the intervention groups (U and E–U) and control mice. *a.u.* arbitrary units, *E–U* elastase–urethane, *OD* optical densities, *PPAR-* peroxisome proliferator-activated receptor alpha; *PPAR-* peroxisome proliferator-activated receptor gamma, *PGC-1* peroxisome proliferator-activated receptor gamma coactivator-1α, *U* urethane

All the analyses of muscle growth and metabolism were carried out in the 35-week cohort mice. Protein levels of myogenin were only significantly decreased in the diaphragm of E–U mice compared to non-exposed controls (Fig. 12a; Additional file 1: Figure S7). In U and E–U mice, protein levels of PPAR-α and PPAR-γ were significantly decreased in the diaphragm compared to control animals, and levels of the latter marker was also significantly reduced in limb muscle of U and E–U mice compared to controls in both time-cohorts (Fig. 12b, c; Additional file 1: Figures S8, S9). Protein levels of PGC-1α did not significantly differ among the study groups in any of the muscles (Fig. 12d; Additional file 1: S10).

### Muscle redox balance proteins

**Fig. 13:**
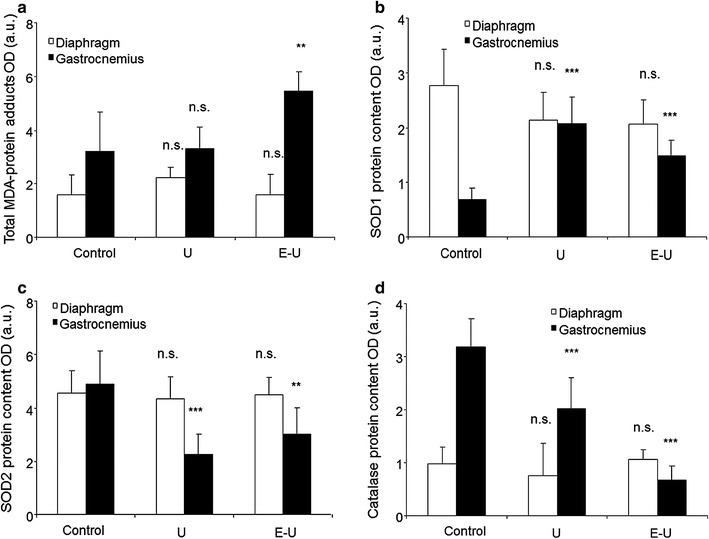
**a** Mean values and standard deviation of MDA, in diaphragm (*white bars*) and gastrocnemius (*black bars*) muscles as measured by optical densities. Statistical significance is represented as follows: (i) n.s.: non-significant and **p ≤ 0.01 between any of the intervention groups (U and E–U) and control mice. **b** Mean values and standard deviation of SOD1, in diaphragm (*white bars*) and gastrocnemius (*black bars*) muscles as measured by optical densities. Statistical significance is represented as follows: n.s.: non-significant and ***p ≤ 0.001 between any of the intervention groups (U and E–U) and control mice. **c** Mean values and standard deviation of SOD2, in diaphragm (*white bars*) and gastrocnemius (*black bars*) muscles as measured by optical densities. Statistical significance is represented as follows: n.s.: non-significant, **p ≤ 0.01 and ***p ≤ 0.001 between any of the intervention groups (U and E–U) and control mice. **d** Mean values and standard deviation of catalase, in diaphragm (*white bars*) and gastrocnemius (*black bars*) muscles as measured by optical densities. Statistical significance is represented as follows: n.s.: non-significant and ***p ≤ 0.001 between any of the intervention groups (U and E–U) and control mice. *a.u.* arbitrary units, *E–U* elastase–urethane, *MDA* malondialdehyde, *OD* optical densities, *SOD1* superoxide dismutase isoform 1, *SOD2* superoxide dismutase isoform 2, *U* urethane

All the analyses of muscle redox balance proteins were carried out in the 35-week cohort mice. Total MDA-protein adducts were only significantly increased in the gastrocnemius of E–U mice compared to non-exposed controls (Fig. 13a; Additional file 1: Figure S11). A significant rise in SOD1 levels was also detected in the limb muscle of both U and E–U groups compared to control mice, while no significant differences were detected in the diaphragm (Fig. 13b; Additional file 1: S12). However, SOD2 and catalase protein levels were significantly reduced in the gastrocnemius of both U and E–U mice compared to non-exposed controls, whereas no significant differences were detected in the diaphragm (Fig. 13c, d; Additional file 1: Figures S13, S14, respectively).

### Muscle inflammation

#### Inflammatory cytokines

**Fig. 14:**
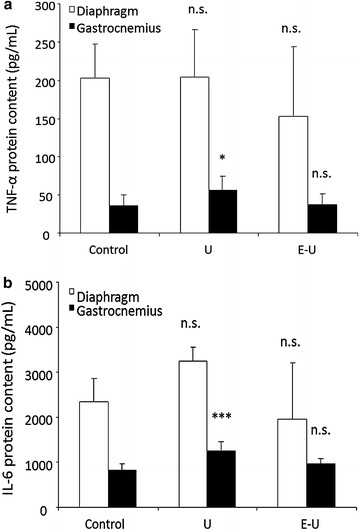
**a** Mean values and standard deviation of TNF-α in diaphragm (*white bars*) and gastrocnemius (*black bars*) muscles as measured by picogram per milliliter (pg/mL). Statistical significance is represented as follows: n.s.: non-significant and *p ≤ 0.05 between any of the intervention groups (U and E–U) and control mice. **b** Mean values and standard deviation of IL-6 in diaphragm (*white bars*) and gastrocnemius (*black bars*) muscles as measured by picogram per milliliter (pg/mL). Statistical significance is represented as follows: n.s.: non-significant and ***p ≤ 0.001 between any of the intervention groups (U and E–U) and control mice. *E–U* elastase–urethane, *IL-6* interleukin-6, *TNF-* tumor necrosis factor-alpha, *U* urethane

In the 35-week cohort of mice, a significant rise in both TNF-α and IL-6 protein levels were seen in the gastrocnemius of U animals compared to non-exposed controls, whereas no significant differences were observed in the diaphragm (Fig. 14a, b).

### Markers of proteolysis in muscles

**Fig. 15:**
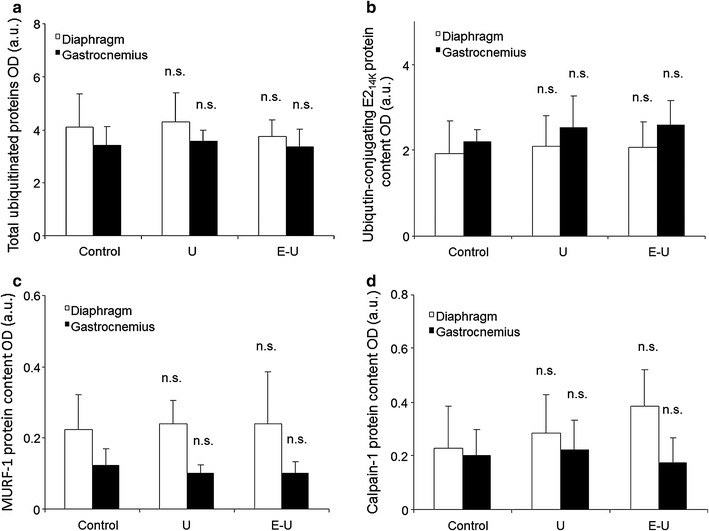
**a** Mean values and standard deviation of total protein ubiquitination in diaphragm (*white bars*) and gastrocnemius (*black bars*) muscles as measured by optical densities. Statistical significance is represented as follows: (i) n.s.: non-significant between any of the intervention groups (U and E–U) and control mice. **b** Mean values and standard deviation of E2_14K_, in diaphragm (*white bars*) and gastrocnemius (*black bars*) muscles as measured by optical densities. Statistical significance is represented as follows: n.s.: non-significant any of the intervention groups (U and E–U) and control mice. **c** Mean values and standard deviation of MURF-1, in diaphragm (*white bars*) and gastrocnemius (*black bars*) muscles as measured by optical densities. Statistical significance is represented as follows: n.s.: non-significant between any of the intervention groups (U and E–U) and control mice. **d** Mean values and standard deviation of calpain-1, in diaphragm (*white bars*) and gastrocnemius (*black bars*) muscles as measured by optical densities. Statistical significance is represented as follows: n.s.: non-significant between any of the intervention groups (U and E–U) and control mice. *a.u.* arbitrary units, *E–U* elastase–urethane, *E2*_*14k*_ ubiquitin-conjugating enzyme E2 (14k), *MURF-1* muscle ring finger protein 1, *OD* optical densities, *U* urethane

All the analyses of proteolytic systems were carried out in the 35-week cohort mice. Protein levels of the following proteolytic markers: total protein ubiquitination, ubiquitin-conjugating E2_14K_, MURF-1, and calpain-1 did not significantly differ among the study groups in either time-cohorts (Fig. 15a–d; Additional file 1: Figures S15–18).

### Markers of autophagy in muscles

**Fig. 16:**
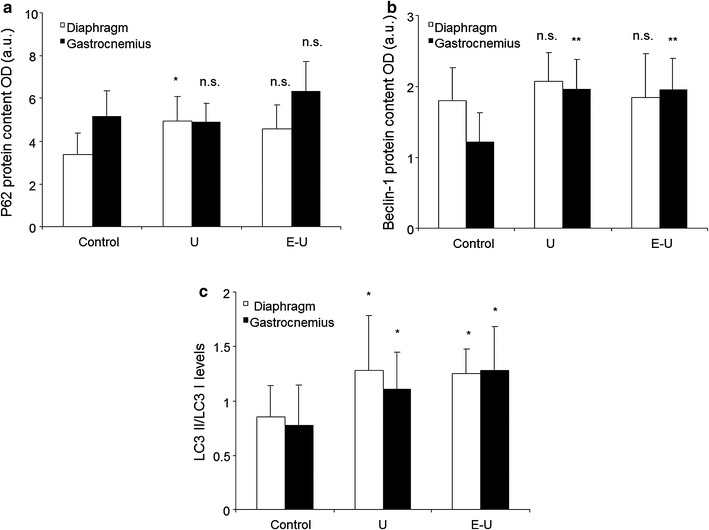
**a** Mean values and standard deviation of p62, in diaphragm (*white bars*) and gastrocnemius (*black bars*) muscles as measured by optical densities. Statistical significance is represented as follows: (i) n.s.: non-significant and *p ≤ 0.05 between any of the intervention groups (U and E–U) and control mice. **b** Mean values and standard deviation of Beclin-1, in diaphragm (*white bars*) and gastrocnemius (*black bars*) muscles as measured by optical densities. Statistical significance is represented as follows: n.s.: non-significant and **p ≤ 0.01 between any of the intervention groups (U and E–U) and control mice. **c** Mean values and standard deviation of LC3 II/LC3 I levels, in diaphragm (*white bars*) and gastrocnemius (*black bars*) muscles as measured by optical densities. Statistical significance is represented as follows: *p ≤ 0.05 between any of the intervention groups (U and E–U) and control mice. *a.u.* arbitrary units, *E–U* elastase–urethane, *LC3* light chain 3, *OD* optical densities, *p62* nucleoporin p62, *U* urethane

All the analyses of muscle autophagy were carried out in the 35-week cohort mice. Protein levels of P62 were only significantly increased in the diaphragm of U mice compared to non-exposed controls (Fig. 16a; Additional file 1: Figure S19). In U and E–U mice, protein levels of beclin-1 and LC3 were significantly increased in the limb muscle compared to the controls (Fig. 16b, c; Additional file 1: Figures S20, S21). Furthermore, in the diaphragm, LC3 levels were also significantly greater in U and E–U mice than in the controls (Fig. 16c; Additional file 1: Figure S21).

## Discussion

Whole body weight and muscle mass loss and the profile of alterations in muscle fiber phenotype and molecular markers of protein metabolism were in general similar in the respiratory and limb muscles of both groups of mice bearing the lung tumors regardless of underlying emphysema. Interestingly, when the relative individual muscle mass to total body weight parameter was estimated in all the experimental groups, significant reductions were observed in diaphragm (both time-cohorts) and gastrocnemius (35-week cohort) only in U mice compared to the non-exposed controls. Body weight gain was significantly more reduced in E–U mice, especially in those of the 35-week cohort. Indeed, actual body weights were of similar values at the end of both 35- and 24-week periods, while control and U animals were of larger size at 35-week than at 24-week time-points. In addition, the cross-sectional area of slow- and fast-twitch muscle fibers was significantly smaller in both diaphragm and gastrocnemius muscles of the tumor-bearing animals, with a significantly larger reduction in the respiratory muscle of the E–U groups in both time-cohorts. In the investigation, other interesting findings were the rise in the number of total muscle abnormalities, including increased inflammatory cells in diaphragm of both time-cohorts, and apoptotic nuclei counts in both muscles studied of the tumor-bearing mice (24- and 35-week time cohorts) regardless of emphysema along with changes in the expression of biological markers involved in muscle mass maintenance and metabolism, which are discussed below.

In the present study, the size of slow- and fast-twitch fibers was significantly reduced in the respiratory and limb muscles of U and E–U mice of both time-cohorts. These findings are in agreement with those reported in previous investigations, in which the cross-sectional area of both types of fibers were also significantly smaller in diaphragm and gastrocnemius muscles of different experimental models of muscle wasting such as chronic heart failure [6] and cancer-induced cachexia [6–8, 11]. Collectively, these results suggest that the process of muscle mass loss associated with chronic conditions equally affects both types of muscles in experimental models of muscle wasting [6–8, 47]. It should also be highlighted that when the parameter muscle weight to total body weight was analyzed, a significant decline in muscle mass was only seen in U group, but not in E–U mice. This is likely due to the fact that the animals in the latter group were of smaller size as determined by their total body weight, thus implying that muscle mass was relatively better preserved in E–U mice compared to U rodents. It is possible that hypoxia, although not measured in the study, may have limited the growth of mice in the E–U group (underlying emphysema) throughout time as shown to occur in other models [48, 49]. Nonetheless, we believe that elucidation of the mechanisms accounting for this observation are beyond the scope of the current study, as muscle mass was, indeed, reduced in both types of muscles in U and E–U groups in the two time-cohorts. On the other hand, the decrease observed in muscle mass loss was probably not due to a decline in food intake as no evident reductions in this parameter or even in the physical activity of the animals were detected during the study period, despite not having been specifically quantified in the investigation. In keeping with the present findings, patients with muscle wasting and cachexia related to chronic conditions also exhibited muscle atrophy, especially of the fast-twitch fibers in their lower limb muscles [4, 16, 18]. These phenotypic features have also been shown in the quadriceps of elderly subjects, in whom the muscle fibers were more prone to experience reductions in their cross-sectional area [50–52].

Levels of proinflammatory cytokines (TNF-α and IL-6) that may mediate muscle wasting [13], although low, were significantly increased in the limb muscle of tumor-bearing animals without underlying emphysema (U group). Furthermore, inflammatory cell counts significantly increased in both types of muscles of the 35-week cohort, as well as in the diaphragm of the 20-week cohort. These mechanisms may explain the relatively larger reduction in muscle mass observed in U group, especially the diaphragm.

Importantly, in both time-cohorts, the greatest reduction in the size of slow- and fast-twitch muscle fibers was achieved in the diaphragm of tumor-bearing mice, especially in those with underlying emphysema. Interestingly, the characteristic switch towards a more fatigue-resistant phenotype identified in the diaphragm of patients with advanced COPD [17, 29] was not observed in the respiratory muscle of the study animals. It is likely that a longer exposure to inspiratory loading is required in order to trigger a switch towards a more fatigue-resistant phenotype of the main respiratory muscle in mice. Enhanced protein catabolism as a result of the increased burden imposed by the underlying respiratory emphysematous condition (increased inspiratory loads) together with the presence of the lung tumors, which further increase the respiratory loads, may explain the smaller cross-sectional area of the diaphragm fibers found in the muscle-wasted mice. In fact, protein levels of specific muscle proteins such as creatine kinase, MyHC and actin were significantly reduced in the diaphragms of U and E–U mice in both time-cohorts. In the gastrocnemius, however, underlying emphysema did not further reduce the size of either slow- or fast-twitch muscle fibers in the cancer cachectic animals, and actin levels in the limb muscle of E–U mice were as high as those detected in the same muscles of control animals. Together these observations point towards a predominant systemic effect of carcinogenesis on muscle mass loss of both diaphragm and gastrocnemius in the urethane-exposed mice along with the local effects imposed by the emphysema-related respiratory loads in the diaphragm muscle.

A significant rise in several structural abnormalities such as inflammatory cell (especially the diaphragm) and internal nuclei counts was also observed in the diaphragm and gastrocnemius muscles of the tumor-bearing mice in the two experimental groups of both study cohorts. The number of apoptotic nuclei was also significantly increased in the respiratory and limb muscles of the same animals in both experimental groups of the two study cohorts. Collectively, these findings are totally in line with those reported in previous investigations, in which the levels of structural abnormalities and those of TUNEL-positive nuclei were also detected in the respiratory and peripheral muscles of animals with muscle wasting associated with several conditions [6–8], as well as in patients with COPD [4, 44] and LC [4]. These results also point towards the existence of a damage-repair mechanism within the myofibers of the target muscles in response to muscle mass loss, which seemed to have taken place in the respiratory and peripheral muscles in a similar fashion. Indeed, the increase in the numbers of inflammatory cells and internal nuclei imply the existence of an underlying repair process following injury of the mouse skeletal muscles within the frame of LC- and emphysema-induced muscle wasting.

Additionally, from a molecular standpoint, alterations in the levels of muscle regeneration and metabolism, namely myogenin, PPAR-α and PPAR-γ were also seen in the diaphragm and gastrocnemius of tumor-bearing mice. PPARs are strong regulators of muscle metabolism and mitochondrial biogenesis [53, 54], and are also involved in the fiber type shift towards a more resistant phenotype [55]. PPARs may also underlie signaling of muscle atrophy in cachectic conditions [19]. In the current study, levels of PPAR-α were significantly decreased in the diaphragm of tumor-bearing mice regardless of emphysema, while a decline in PPAR-gamma levels was identified in both respiratory and limb muscles of the same groups of animals. These are interesting observations that are in accordance with a previous investigation, in which similar findings were also reported in the vastus lateralis of patients with severe COPD, especially in those with muscle wasting [19]. Taken together, it is possible to conclude that PPARs underlie atrophy signaling in both respiratory and peripheral muscles, at least in these models of cachexia. Nevertheless, levels of proteolytic markers (ubiquitin–proteasome system) were not modified in the muscles of either U or E–U mice in any of the study cohorts compared to non-exposed controls. The rise in apoptotic nuclei and autophagy markers were probably the most relevant contributors to the atrophy observed in the myofibers of the diaphragm and gastrocnemius muscles of the cachectic mice in both time-cohorts as was also shown to occur in previous studies [6–8].

Levels of protein oxidation were significantly increased in the gastrocnemius muscle of E–U cachectic mice, but not in U animals. These results are in line with those reported in our previous study, in which protein oxidation levels also increased in the muscles of mice with elastase-induced emphysema [9], as well as in other investigations conducted on COPD patients, in whom oxidative stress levels were also increased in their respiratory [17, 29] and limb muscles [4, 25–27, 31, 56–58]. Furthermore, in both experimental mouse groups, protein levels of mitochondrial SOD2 and catalase were significantly decreased only in the gastrocnemius but not the diaphragm, while those of the cytosolic SOD1 were significantly increased. These results are partly in keeping with those shown in the diaphragm of the elastase-induced emphysema mice [9], and in those of the vastus lateralis of COPD patients, in whom SOD1 protein levels were also increased [4]. The degree of muscle mass loss together with differences in the study models may account for the discrepancies observed in antioxidant enzyme levels among studies.

### Study limitations

The current model of induction of emphysema has been well-validated in the literature as many publications are currently available in which elastase has been used to induce emphysema in animal models [59, 60]. In the present investigation, elastase was chosen as it induces a rapid onset of emphysema in the lungs. Indeed, emphysema developed at a much earlier stage than did the tumors in the mouse lungs, and this was a major objective in the study. A limitation in the investigation was the lack of functional data from the study muscles. However, as the current investigation represents a first approach to confirm the main hypothesis, we believe that this is not a major limitation, since it has been clearly confirmed by the histological and molecular experiments conducted in the investigation that muscle mass loss and atrophy occur in both respiratory and limb muscles in this model of lung carcinogenesis-induced muscle wasting. Moreover, experimental mouse models of cancer-induced cachexia are not characterized by the loss of body and/or muscle mass as shown to occur in actual patients, since the animals are still growing. Therefore, we believe that the reduction in both body weight gain and muscle sizes at the end of study protocol were the variables that best represented the decrease in muscle mass observed in the tumor-bearing animals with and without emphysema. Indeed, this is a general limitation in any animal model of cachexia and muscle wasting.

## Conclusions

In this mouse model of lung carcinogenesis with and without emphysema, reduced body weight gain, and muscle mass loss and atrophy were indeed observed in the respiratory and limb muscles of the animals after 20- and 35-week exposure times. In the mice, the process of muscle wasting was characterized by a rise in the number of inflammatory cells, internal nuclei, apoptotic nuclei, and autophagy markers, but not proteolysis, while a decrease in the content of contractile proteins, creatine kinase, antioxidant enzymes, and PPARs was observed in a similar fashion in both respiratory and limb muscles. Nevertheless, underlying emphysema induced a larger reduction in the size of slow- and fast-twitch fibers in the diaphragm of tumor-bearing mice probably as a result of the greater inspiratory burden imposed onto this muscle. These findings should be taken into consideration when evaluating muscle wasting in patients with lung cancer cachexia with and without underlying emphysema.

## Additional file

10.1186/s12967-016-1003-9 Online data supplement.

## Abbreviations

ANOVA: one-way analysis of variance
ATP: adenosine triphosphate
CIMA: Center for Applied Medical Research
COPD: chronic obstructive pulmonary disease
CT: computed tomography
E: elastase
E2: ubiquitin-conjugating enzyme 2
ELISA: enzyme-linked immunosorbent assay
E–U: elastase–urethane
GAPDH: glyceraldehyde-3-phosphate dehydrogenase
HRP: horseradish peroxidase
IL: interleukin
LC3: light chain 3
LC: lung cancer
MDA: malondialdehyde
MURF: muscle ring finger
MyHC: myosin heavy chain
P62: nucleoporin p62
PGC: PPAR gamma coactivator
PPAR: peroxisome proliferator-activated receptor
SOD: superoxide dismutase
SPSS: Statistical Package for the Social Sciences
TUNEL: terminal deoxynucleotidyl transferase- mediated uridine 5′-triphosphate (UTP) nick- end labelling
TNF: tumor necrosis factor
U: urethane

## References

[1] Anker SD, Ponikowski P, Varney S, Chua TP, Clark AL, Webb-Peploe KM (1997). Wasting as independent risk factor for mortality in chronic heart failure. Lancet.

[2] Anker SD, Sharma R (2002). The syndrome of cardiac cachexia. Int J Cardiol.

[3] Muscaritoli M, Bossola M, Aversa Z, Bellantone R, Rossi FF (2006). Prevention and treatment of cancer cachexia: new insights into an old problem. Eur J Cancer.

[4] Puig-Vilanova E, Rodriguez DA, Lloreta J, Ausin P, Pascual-Guardia S, Broquetas J (2015). Oxidative stress, redox signaling pathways, and autophagy in cachectic muscles of male patients with advanced COPD and lung cancer. Free Radic Biol Med.

[5] Fearon K, Strasser F, Anker SD, Bosaeus I, Bruera E, Fainsinger RL (2011). Definition and classification of cancer cachexia: an international consensus. Lancet Oncol.

[6] Barreiro E, Puig-Vilanova E, Marin-Corral J, Chacon-Cabrera A, Salazar-Degracia A, Mateu X (2015). Therapeutic approaches in mitochondrial dysfunction, proteolysis, and structural alterations of diaphragm and gastrocnemius in rats with chronic heart failure. J Cell Physiol.

[7] Chacon-Cabrera A, Fermoselle C, Urtreger AJ, Mateu-Jimenez M, Diament MJ, de Kier Joffe ED (2014). Pharmacological strategies in lung cancer-induced cachexia: effects on muscle proteolysis, autophagy, structure, and weakness. J Cell Physiol.

[8] Chacon-Cabrera A, Fermoselle C, Salmela I, Yelamos J, Barreiro E (2015). MicroRNA expression and protein acetylation pattern in respiratory and limb muscles of Parp-1(−/−) and Parp-2(−/−) mice with lung cancer cachexia. Biochim Biophys Acta.

[9] Fermoselle C, Sanchez F, Barreiro E (2011). Reduction of muscle mass mediated by myostatin in an experimental model of pulmonary emphysema. Arch Bronconeumol.

[10] Fermoselle C, Garcia-Arumi E, Puig-Vilanova E, Andreu AL, Urtreger AJ, de Kier Joffe ED (2013). Mitochondrial dysfunction and therapeutic approaches in respiratory and limb muscles of cancer cachectic mice. Exp Physiol.

[11] Marin-Corral J, Fontes CC, Pascual-Guardia S, Sanchez F, Olivan M, Argiles JM (2010). Redox balance and carbonylated proteins in limb and heart muscles of cachectic rats. Antioxid Redox Signal.

[12] Argiles JM, Lopez-Soriano FJ (1996). The ubiquitin-dependent proteolytic pathway in skeletal muscle: its role in pathological states. Trends Pharmacol Sci.

[13] Argiles JM, Busquets S, Lopez-Soriano FJ (2011). Anti-inflammatory therapies in cancer cachexia. Eur J Pharmacol.

[14] Fontes-Oliveira CC, Busquets S, Toledo M, Penna F, Paz AM, Sirisi S (2013). Mitochondrial and sarcoplasmic reticulum abnormalities in cancer cachexia: altered energetic efficiency?. Biochim Biophys Acta.

[15] Penna F, Baccino FM, Costelli P (2014). Coming back: autophagy in cachexia. Curr Opin Clin Nutr Metab Care.

[16] Fermoselle C, Rabinovich R, Ausin P, Puig-Vilanova E, Coronell C, Sanchez F (2012). Does oxidative stress modulate limb muscle atrophy in severe COPD patients?. Eur Respir J.

[17] Marin-Corral J, Minguella J, Ramirez-Sarmiento AL, Hussain SN, Gea J, Barreiro E (2009). Oxidised proteins and superoxide anion production in the diaphragm of severe COPD patients. Eur Respir J.

[18] Puig-Vilanova E, Martinez-Llorens J, Ausin P, Roca J, Gea J, Barreiro E (2015). Quadriceps muscle weakness and atrophy are associated with a differential epigenetic profile in advanced COPD. Clin Sci (Lond).

[19] Remels AH, Schrauwen P, Broekhuizen R, Willems J, Kersten S, Gosker HR (2007). Peroxisome proliferator-activated receptor expression is reduced in skeletal muscle in COPD. Eur Respir J.

[20] Remels AH, Gosker HR, Schrauwen P, Hommelberg PP, Sliwinski P, Polkey M (2010). TNF-α impairs regulation of muscle oxidative phenotype: implications for cachexia?. FASEB J.

[21] Maltais F, Simard AA, Simard C, Jobin J, Desgagnes P, LeBlanc P (1996). Oxidative capacity of the skeletal muscle and lactic acid kinetics during exercise in normal subjects and in patients with COPD. Am J Respir Crit Care Med.

[22] Maltais F, LeBlanc P, Whittom F, Simard C, Marquis K, Belanger M (2000). Oxidative enzyme activities of the vastus lateralis muscle and the functional status in patients with COPD. Thorax.

[23] Maltais F, Decramer M, Casaburi R, Barreiro E, Burelle Y, Debigare R (2014). An Official American Thoracic Society/European Respiratory Society Statement: update on limb muscle dysfunction in chronic obstructive pulmonary disease. Am J Respir Crit Care Med.

[24] Ceelen JJ, Langen RC, Schols AM (2014). Systemic inflammation in chronic obstructive pulmonary disease and lung cancer: common driver of pulmonary cachexia?. Curr Opin Support Palliat Care.

[25] Barreiro E, Schols AM, Polkey MI, Galdiz JB, Gosker HR, Swallow EB (2008). Cytokine profile in quadriceps muscles of patients with severe COPD. Thorax.

[26] Barreiro E, Peinado VI, Galdiz JB, Ferrer E, Marin-Corral J, Sanchez F (2010). Cigarette smoke-induced oxidative stress: a role in chronic obstructive pulmonary disease skeletal muscle dysfunction. Am J Respir Crit Care Med.

[27] Barreiro E, Bustamante V, Cejudo P, Galdiz JB, Gea J, de Lucas P (2015). Guidelines for the evaluation and treatment of muscle dysfunction in patients with chronic obstructive pulmonary disease. Arch Bronconeumol.

[28] Guo Y, Gosker HR, Schols AM, Kapchinsky S, Bourbeau J, Sandri M (2013). Autophagy in locomotor muscles of patients with chronic obstructive pulmonary disease. Am J Respir Crit Care Med.

[29] Barreiro E, de la Puente B, Minguella J, Corominas JM, Serrano S, Hussain SN (2005). Oxidative stress and respiratory muscle dysfunction in severe chronic obstructive pulmonary disease. Am J Respir Crit Care Med.

[30] Puig-Vilanova E, Aguilo R, Rodriguez-Fuster A, Martinez-Llorens J, Gea J, Barreiro E (2014). Epigenetic mechanisms in respiratory muscle dysfunction of patients with chronic obstructive pulmonary disease. PLoS One.

[31] Barreiro E, Gea J, Matar G, Hussain SN (2005). Expression and carbonylation of creatine kinase in the quadriceps femoris muscles of patients with chronic obstructive pulmonary disease. Am J Respir Cell Mol Biol.

[32] Artaechevarria X, Blanco D, de Biurrun G, Ceresa M, Perez-Martin D, Bastarrika G (2011). Evaluation of micro-CT for emphysema assessment in mice: comparison with non-radiological techniques. Eur Radiol.

[33] Marcos JV, Munoz-Barrutia A, Ortiz-de-Solorzano C, Cristobal G (2015). Quantitative assessment of emphysema severity in histological lung analysis. Ann Biomed Eng.

[34] Rudyanto RD, Bastarrika G, de Biurrun G, Agorreta J, Montuenga LM, Ortiz-de-Solorzano C (2013). Individual nodule tracking in micro-CT images of a longitudinal lung cancer mouse model. Med Image Anal.

[35] de-Torres JP, Wilson DO, Sanchez-Salcedo P, Weissfeld JL, Berto J, Campo A (2015). Lung cancer in patients with chronic obstructive pulmonary disease. Development and validation of the COPD lung cancer screening score. Am J Respir Crit Care Med.

[36] Leiro-Fernandez V, Mouronte-Roibas C, Ramos-Hernandez C, Botana-Rial M, Gonzalez-Pineiro A, Garcia-Rodriguez E (2014). Changes in clinical presentation and staging of lung cancer over two decades. Arch Bronconeumol.

[37] Miravitlles M, Soler-Cataluna JJ, Calle M, Molina J, Almagro P, Quintano JA (2014). Spanish guideline for COPD (GesEPOC). Update 2014. Arch Bronconeumol.

[38] Sanchez-Salcedo P, Berto J, de-Torres JP, Campo A, Alcaide AB, Bastarrika G (2015). Lung cancer screening: fourteen year experience of the Pamplona early detection program (P-IELCAP). Arch Bronconeumol..

[39] Sanchez-Salcedo P, Wilson DO, de-Torres JP, Weissfeld JL, Berto J, Campo A (2015). Improving selection criteria for lung cancer screening. The potential role of emphysema. Am J Respir Crit Care Med.

[40] Miravitlles M, Worth H, Soler-Cataluna JJ, Price D, De Benedetto F, Roche N, et al. The relationship between 24-hour symptoms and COPD exacerbations and healthcare resource use: results from an observational study (ASSESS). COPD. 2016;1–8. doi: 10.3109/15412555.2016.1150447. 10.3109/15412555.2016.1150447 26983349

[41] Miravitlles M (2016). What was the impact of the Spanish COPD guidelines (GesEPOC) and how can they be improved?. Arch Bronconeumol.

[42] Costelli P, Muscaritoli M, Bossola M, Penna F, Reffo P, Bonetto A (2006). IGF-1 is downregulated in experimental cancer cachexia. Am J Physiol Regul Integr Comp Physiol.

[43] Munoz-Barrutia A, Ceresa M, Artaechevarria X, Montuenga LM, Ortiz-de-Solorzano C (2012). Quantification of lung damage in an elastase-induced mouse model of emphysema. Int J Biomed Imaging.

[44] Barreiro E, Ferrer D, Sanchez F, Minguella J, Marin-Corral J, Martinez-Llorens J (2011). Inflammatory cells and apoptosis in respiratory and limb muscles of patients with COPD. J Appl Physiol.

[45] Busquets S, Figueras MT, Fuster G, Almendro V, Moore-Carrasco R, Ametller E (2004). Anticachectic effects of formoterol: a drug for potential treatment of muscle wasting. Cancer Res.

[46] Busquets S, Serpe R, Toledo M, Betancourt A, Marmonti E, Orpi M (2012). l-Carnitine: an adequate supplement for a multi-targeted anti-wasting therapy in cancer. Clin Nutr.

[47] Roberts BM, Ahn B, Smuder AJ, Al-Rajhi M, Gill LC, Beharry AW (2013). Diaphragm and ventilatory dysfunction during cancer cachexia. FASEB J.

[48] de Theije CC, Langen RC, Lamers WH, Gosker HR, Schols AM, Kohler SE (2015). Differential sensitivity of oxidative and glycolytic muscles to hypoxia-induced muscle atrophy. J Appl Physiol (1985).

[49] Slot IG, Schols AM, de Theije CC, Snepvangers FJ, Gosker HR (2016). Alterations in skeletal muscle oxidative phenotype in mice exposed to 3 weeks of normobaric hypoxia. J Cell Physiol.

[50] Lexell J, Taylor CC, Sjostrom M (1988). What is the cause of the ageing atrophy? Total number, size and proportion of different fiber types studied in whole vastus lateralis muscle from 15- to 83-year-old men. J Neurol Sci.

[51] Lexell J, Downham D (1992). What is the effect of ageing on type 2 muscle fibres?. J Neurol Sci.

[52] Lexell J (1995). Human aging, muscle mass, and fiber type composition. J Gerontol A Biol Sci Med Sci.

[53] Koves TR, Li P, An J, Akimoto T, Slentz D, Ilkayeva O (2005). Peroxisome proliferator-activated receptor-gamma co-activator 1α-mediated metabolic remodeling of skeletal myocytes mimics exercise training and reverses lipid-induced mitochondrial inefficiency. J Biol Chem.

[54] Luquet S, Lopez-Soriano J, Holst D, Fredenrich A, Melki J, Rassoulzadegan M (2003). Peroxisome proliferator-activated receptor delta controls muscle development and oxidative capability. FASEB J.

[55] Puigserver P, Spiegelman BM (2003). Peroxisome proliferator-activated receptor-gamma coactivator 1α (PGC-1α): transcriptional coactivator and metabolic regulator. Endocr Rev.

[56] Couillard A, Maltais F, Saey D, Debigare R, Michaud A, Koechlin C (2003). Exercise-induced quadriceps oxidative stress and peripheral muscle dysfunction in patients with chronic obstructive pulmonary disease. Am J Respir Crit Care Med.

[57] Koechlin C, Couillard A, Simar D, Cristol JP, Bellet H, Hayot M (2004). Does oxidative stress alter quadriceps endurance in chronic obstructive pulmonary disease?. Am J Respir Crit Care Med.

[58] Rodriguez DA, Kalko S, Puig-Vilanova E, Perez-Olabarria M, Falciani F, Gea J (2012). Muscle and blood redox status after exercise training in severe COPD patients. Free Radic Biol Med.

[59] Jiang Z, Lao T, Qiu W, Polverino F, Gupta K, Guo F (2016). A chronic obstructive pulmonary disease susceptibility gene, FAM13A, regulates protein stability of β-catenin. Am J Respir Crit Care Med.

[60] Kai Y, Tomoda K, Yoneyama H, Yoshikawa M, Kimura H (2015). RNA interference targeting carbohydrate sulfotransferase 3 diminishes macrophage accumulation, inhibits MMP-9 expression and promotes lung recovery in murine pulmonary emphysema. Respir Res.

