# Supplementary material for: Phenotypic and metabolic features of mouse diaphragm and gastrocnemius muscles in chronic lung carcinogenesis: influence of underlying emphysema
Source: J Transl Med. 2016 Aug 23;14(1):244. doi: 10.1186/s12967-016-1003-9 (PMC4994253; doi:10.1186/s12967-016-1003-9)

**Online data supplement**

**PHENOTYPIC AND METABOLIC FEATURES of MOUSE diaphragm and gastrocnemius MUSCLES IN CHRONIC lung caRCINOGENESIS: INFLUENCE OF UNDERLYING EMPHYSEMA**

**Anna Salazar-Degracia,** **David Blanco, Mònica Vilà-Ubach, Gabriel de Biurrun, Carlos Ortiz de Solórzano, Luis M. Montuenga, Esther Barreiro**

**MATERIALS AND METHODS**

**Animal experiments**

*Experimental design.* Forty-eight male A/J strain mice (2-months old, 16-23g) were used for the purpose of the investigation, which were randomly divided into two independent time-cohorts: 20 weeks and 35 weeks. Animals were further subdivided into the following groups in each time-cohort (N=8/group): 1) non-exposed control mice, 2) lung carcinogenesis mice induced by urethane (U group), and 3) lung emphysema-carcinogenesis mice induced by elastase and urethane (E-U group). Lung carcinogenesis was induced as a result of a single intraperitoneal injection of 1 mg/g urethane (Urethane U2500 Sigma, St. Louis, Missouri, USA). Lung emphysema was induced through a single oropharyngeal instillation of 6 units per 30 g body weight of porcine pancreatic elastase (PPE, EC134GI, EPC, MI, USA) 0.15 mg/100 g elastase-high purity (EC134GI, Elastin Products Company, Owensville, Missouri, USA), following previously reported methodologies (1;2).

*Cancer and emphysema induction protocols.* Briefly, after administration of 2% isoflurane in an induction box, the animals were placed on a ramp with an angle of 60º. Once immobilized, the volume of elastase was deposited in their mouth. The nostrils were occluded to force breathing through the mouth, thus enabling the mice to inhale the solution. After aspiration, the animals rested on the ramp for a few seconds to let them recover the normal breathing. Animals exposed to both emphysema and lung carcinogenesis received an oropharyngeal instillation of elastase on day 0 together with a single intraperitoneal injection of urethane on day 9 (Figure 1A). The lung carcinogenesis group (U) received an oropharyngeal instillation of saline solution on day 0 and a single intraperitoneal injection of urethane on day 9 (Figure 1B). Control groups were followed up for 20 or 35 weeks depending on the experimental time-cohort. These animals received oropharyngeal or intraperitoneal injections of saline solutions (0.1 mL) on days 0 or 9, respectively (Figure 1C), as also described in previous investigations (1-3)**.** The different times-cohorts (20- and 35-weeks) started on day 9 after the urethane or saline (controls) injection in all the study groups.

This study was designed in accordance with the ethical regulations on animal experimentation (EU 2010/63 CEE, *Real Decreto* 53/2013 BOE 34, Spain) at the Center for Applied Medical Research (CIMA), and the WMA Declaration of Helsinki in what respects to the use of animals for research. Ethical approval was obtained by the Animal Experimentation Ethics Committee of CIMA in Pamplona (Spain), where the animal experiments were all conducted together with the lung histological analyses.

**In vivo measurements conducted on the animals**

Water and food were administered to the animals for the entire duration of the study and they remained under housing environmental conditions in a 12:12 light-dark cycles. In all the mice, body weight was determined on day 0 before treatment administration and at 4- and 14-week time points, and immediately prior to their sacrifice at either 20- or 35- week time-points in each cohort (1;2). The percentage of body weight gain at the end of the study period was calculated as follows: [(body weight on either week 35 or 24 – body weight on day 0)] / body weight on day 0 x 100. Additionally, the percentage of muscle mass relative to final body weight in each animal was calculated as follows: [(muscle mass weight / final body weight) x 100].

*Micro-computer tomography (CT).* Prior to sacrifice, animals underwent CT scan analyses from their lungs, which were used to verify the presence of the emphysema with and without the tumors following previously reported methodologies (1;3). Before the scan, animals were intraperitoneally anaesthetized prior to intubation, to be then connected to a Flexivent rodent ventilator (Scireq, Montreal, Canada). Animals were kept alive (breathing), under anesthesia (2% isoflurane) until complete relaxation was reached. During the scan analyses, 0.5% isoflurane was administered to the mice. All mice were scanned with an X-ray micro-CT (Micro-CAT II, Siemens Pre-Clinical Solutions, Knoxville, Tennessee, USA), with a source 80 voltage kV and a 500 microA current. Seven hundred micro-CT projections were acquired during isopressure breaths, which were hold for 650 milliseconds (450 millisecond-exposure time/ projection). A commercially available Dose Calculator software (Siemens Pre-clinical Solutions, Knoxville, Tennessee, USA) was used to estimate X-ray dosage-computed. In the current investigation, specific measurements of the degree of emphysema were not carried out in the animals as these analyses had been conducted in previously published studies and the model was extremely reproducible (1;3-5) .

Prior to sacrifice an intraperitoneal injection of 90 mg/kg ketamine (Imalgène®, Merial, Lyon, France) and 10 mg/kg xylazine (Rompun®, Bayer AG, Leverkusen, Germany) was administered. In all cases, the pedal and blink reflexes were evaluated in order to verify total anesthesia depth. Animals were exsanguinated to facilitate removal or organs and skeletal muscles. Immediately afterwards, in all groups of mice, the lungs, the diaphragm and gastrocnemius muscles were obtained: gastrocnemius was obtained in the first place while the animals were still breathing, subsequently to collect the diaphragm and lungs while the mice were still alive as conducted in previous studies (1;2;6;7)**.** All muscle specimens of the 20-week cohort were immersed in buffered 4% formaldehyde for 24 hours and washed in several alcohol steps prior to paraffin-embedding. In the 35-week cohort of animals, the muscles, which were of larger size, were split in two samples, one of which was also immediately fixed in 4% formaldehyde, rinsed in alcohols and embedded in paraffin, and the rest of the tissue was immediately frozen in liquid nitrogen to be subsequently preserved at -80ºC until further use. Paraffin-embedded tissues were used to analyze the histological features of tumors in the lungs, while in the muscles, fiber types, potential structural abnormalities, and apoptotic nuclei counts were determined. Moreover, frozen muscle specimens were used for immunoblotting and enzyme-linked immunoabsorbent assay (ELISA) procedures.

**Molecular biology analyses**

All biological analyses were performed in the same laboratory at *IMIM-Hospital del Mar* (Barcelona, Spain) except for the lung histological analyses that were performed at CIMA (Pamplona, Spain).

*Histological analyses of the lungs.* Lung carcinogenesis histology was evaluated on three micrometer paraffin-embedded sections of the lungs in U and E-U groups of mice, according to previously published methodologies (1;3-5). Lobe sections were stained with hematoxylin and eosin (H&E). Images of the sections were taken at 50x and 200x under light microscopy (Zeis Axioplan 2ie microscope, Carl Zeiss, Jena, Germany).

*Muscle fiber counts and morphometry.* Diaphragm and gastrocnemius muscle fibers were identified on three micrometer thick paraffin-embedded sections in all groups of mice and the two time-cohorts. MyHC-I and MyHC-II isoforms were identified using anti-MyHC-I (clone MHC, Biogenesis Inc., Poole, England, UK) and anti-MyHC-II antibodies (clone MY-32, Sigma, Saint Louis, MO), respectively, according to previously published methodologies (6-10). Images of the muscle cross sections were taken at 200x under light microscopy (Olympus BX 61, Olympus Corporation, Tokyo, Japan) coupled with and image-digitizing camera (Olympus DP 71, Olympus Corporation), using the Imaging Cell-B software (Olympus corporation). The cross-sectional area, mean least diameter, and proportions of type I and type II fibers were assessed using ImageJ software (National Institute of Health, available at <http://rsb.info.nih.gov/ij/>). At least 100 fibers were measured and counted in each sample from both diaphragm and gastrocnemius muscles in all study groups.

*Muscle structural abnormalities.* The area fraction of normal and abnormal muscle was evaluated on three micrometer paraffin-embedded sections of the diaphragm and gastrocnemius muscles in all groups of mice following previously published methodologies (6-8;10;11). Briefly, to determine the proportion of structural abnormalities in normal and pathological tissues, quantitative analysis was performed using computer-assisted point morphometric techniques in all the muscle sections, which had been previously stained with hematoxylin-eosin. A grid of 63 point-intercepts (7 x 9 regular pattern), built by means of the software Imaging Cell-B (Olympus Corporation), was superimposed onto the images of the muscle cross sections at 400x, under light microscopy (Olympus BX 61, Olympus Corporation) using and image digitizing camera (Olympus DP 71, Olympus Corporation). Each point-intercept was assigned to a specific category and entered into the software. Categories for structural abnormalities scoring were defined as follows: 1) normal muscle, 2) internal nuclei, 3) inflammatory cells, 4) lipofuscin, 5) abnormal viable, 6) inflamed/necrotic cells, 7) vessels, and 0) no count. The area fraction for each category was defined as the percentage of points that fell onto each of those characteristics relative to the total number of points superimposed on all viable fields (all features except for categories 0 and 7) of each cross section. The area fraction of normal muscle was equivalent to the proportions of points falling in category 1, while the area fraction of abnormal muscle was determined by calculation of the proportion of points included in the other categories (categories 2 to 6).

*Terminal deoxynucleotidyl transferase- mediated uridine 5’-triphosphate (UTP) nick- end labelling (TUNEL) assay.* In muscle paraffin-embedded sections, apoptotic nuclei were identified using the TUNEL assay (In Situ Cell Death Detection Kit, POD, Roche Applied Science, Mannheim, Germany) in both diaphragm and gastrocnemius muscle specimens from all study groups following the manufacturer’s instructions and previous studies (6-8;12). Briefly, this assay is based on the principal that during apoptotic nuclei genomic DNA may yield double-stranded, low molecular weight fragments as well as single strand breaks (nicks) in high molecular weight DNA. This DNA strand breaks can be identified by labelling 3’-hydroxyl (3’OH) groups with modified nucleotides in an enzymatic reaction. In this assay, deoxynucleotidyl transferase (TdT), which catalyzes the polymerization of nucleotides to free 3’-OH DNA ends, is used to label DNA strand breaks. Briefly, diaphragm and gastrocnemius muscle sections were fixed and permeabilized. Subsequently, they were incubated with the TUNEL reaction mixture that contains terminal TdT and fluorescein-dUTP. During the incubation period, terminal TdT catalyzed the addition of fluorescein-dUTP at free 3’-OH groups in single- and double- stranded DNA. After washing, the label incorporated at the damaged sites of the DNA was marked by anti-fluorescein antibody conjugated with the reporter enzyme peroxidase. After several washes that removed unbound enzyme conjugate, the peroxidase retained in the immune complex was visualized by a substrate reaction. Apoptotic nuclei were brown, while negative nuclei were blue (hematoxylin counterstaining). TUNEL-positive nuclei were those clearly located within the muscle fiber boundary in each section. In each muscle cross-section, the TUNEL-positive nuclei and the total number of nuclei were counted blindly by 2 independent observers, who were previously trained for that purpose. On this basis, in each muscle preparation, altered fibers were expressed as the ratio of total TUNEL positively-stained nuclei to the total number of counted nuclei, as also previously described (6-8;12). A minimum amount of 300 nuclei were counted in each muscle preparation. Final results corresponded to the mean value of the counts provided by the 2 independent observers (concordance rate 95%). Negative control experiments, in which the TUNEL reaction mixture was omitted, were also conducted. Moreover, rat testicles were used as a positive control in these experiments.

*Immunoblotting of 1D electrophoresis.* Protein levels of the different molecular markers explored in the study muscles were analyzed in the diaphragm and gastrocnemius of the experimental groups belonging to the 35-week cohort using previously published methodologies (6-8;12). Briefly, frozen muscle samples from the diaphragm and gastrocnemius of the experimental groups belonging to the 35-week cohort were homogenized in a specific buffer containing 50 mM 4-(2-hydroxyethyl)-1-piperazineethanesulfonic acid (HEPES), 150 mM NaCl, 100 nM NaF, 10 mM Na pyrophosphate, 5 mM ethylenediaminetetraacetic acid (EDTA), 0.5%Triton-X, 2 microg/mL leupeptin, 100 microg/mL phenylmethylsulfonyl fluoride (PMSF), 2 microg/mL aprotinin, and 10 microg/mL pepstatin A. The entire procedures were always conducted at 4ºC (on ice). Protein levels in crude muscle homogenates were spectophotometrically determined using the Bradford method in triplicates for all the samples and bovine serum albumin (BSA) was used as the standard (Bio-Rad protein reagent, Bio-Rad Inc., Hercules, CA, USA). The final protein concentration in each sample was calculated from at least two Bradford measurements that were almost identical. Equal amounts of total protein (ranging from 5 to 20 micrograms, depending on the antigen and antibody) from crude muscle homogenates were always loaded onto the gels, as well as identical sample volumes/lane. In order to carry out the comparisons among the different experimental groups, muscle sample specimens were always run together and kept in the same order. Two independent sets of immunoblots were conducted in which diaphragm and gastrocnemius muscle specimens were run separately. Experiments were confirmed at least twice for all the antigens analyzed in the study. Fresh gels were specifically loaded for each of the antigens in all cases.

Proteins were then separated by electrophoresis, transferred to polyvinylidene difluoride (PVDF) membranes, blocked with 5% non-fat milk or with 1% bovine serum albumin (BSA), depending on the primary antibody and incubated with the corresponding selective primary antibodies overnight. Protein content of the different markers was identified using specific primary antibodies: actin (anti-alpha-sarcomeric actin antibody, clone 5C5, Sigma Sigma-Aldrich, St. Louis, MO, USA), myosin heavy chain (anti-MyHC antibody, clone A4.1025, Upstate-Millipore, Temecula, CA, USA), creatine kinase (anti-creatine kinase antibody, Santa Cruz), carbonic anhydrase-3 (anti-carbonic anhydrase-3 antibody, Santa Cruz Biotechnology, Santa Cruz, CA, USA), myogenin (anti-myogenin antibody, Santa Cruz), catalase (anti-catalase antibody, Calbiochem, Darmstadt, Germany), superoxide dismutase (SOD)2 (anti-SOD2 antibody, Santa Cruz), SOD1 (anti-SOD1 antibody, Santa Cruz), malondialdehyde (MDA) protein adducts (anti-MDA protein adducts antibody Academy Bio-Medical Company, Inc., Houston, TX, USA), peroxisome proliferator-activated receptor (PPAR)-alpha (anti-PPAR-alpha antibody (H-98), Santa Cruz), PPAR-gamma (anti- PPAR-gamma antibody (H-100), Santa Cruz), PPAR gamma coactivator (PGC) -1 alpha (anti-PGC-1 alpha antibody (H-300), Santa Cruz), total protein ubiquitination (anti-protein ubiquitination antibody, Boston Biochem, Cambridge, MA, USA), ubiquitin-conjugating enzyme E2_14k_ (anti-E2_14K_ antibody, Boston Biochem), muscle ring finger (MURF)-1 (anti-MURF-1 antibody, Santa Cruz Biotechnology, Santa Cruz, CA, USA), calpain-1 (anti-calpain-1 antibody, Cell Signaling, USA), nucleoporin p-62 (anti-p62/SQSTM1 antibody, Sigma-Aldrich, St. Louis, MO, USA), beclin-1 (anti-beclin-1 antibody, Santa Cruz), light chain (LC)3B (anti-LC3B antibody, Cell Signaling, Boston, MA, USA), and glyceraldehyde-3-phosphate dehydrogenase (GAPDH, anti-GAPDH antibody, Santa Cruz). Antigens from all the samples were detected using horseradish peroxidase (HRP)-conjugated secondary antibodies (Jackson ImmunoResearch Inc, West Grove, PA, USA) and a chemiluminescence kit (Thermo Scientific, Rockford, IL, USA).

PVDF membranes were scanned with the Molecular Imager Chemidoc XRS System (Bio-Rad Laboratories, Hercules, CA, USA) using the software Quantity One version 4.6.5 (Bio-Rad Laboratories). For each of the study antigens, PVDF membranes of samples from the different groups were always detected in the same picture under identical exposure times. Optical densities of specific proteins were quantified using the software Image Lab version 2.0.1 (Bio-Rad Laboratories). Final optical densities obtained in each specific group of subjects corresponded to the mean values of the different samples (lanes) of each of the study antigens. To validate equal protein loading across lanes, SDS-PAGE gels were stained with Commassie blue, and the glycolytic enzyme GAPDH was used as the protein loading control in all the immunoblots as no significant differences were detected in the levels of expression of this marker across lanes in all the immunoblots (Figures E1 and E2, respectively).

Standard stripping methodologies were employed to detect the protein loading control GAPDH for each of the markers. Briefly, membranes were stripped of primary and secondary antibodies through incubation with a stripping solution (25nM glycine, pH 2.0, and 1% SDS) for 30 minutes, followed by two consecutive washes containing phosphate buffered saline with tween (PBST) at room temperature for 10 minutes. Subsequently, membranes were blocked with either 5% non-fat milk or 1% BSA, depending on the primary antibody and were reincubated with primary and secondary antibodies following the procedures described above.

*Cytokine ELISA*. Protein levels of the inflammatory cytokines tumor necrosis factor (TNF)-alpha and interleukin (IL)-6 were quantified in the diaphragm and gastrocnemius muscles in all study groups of the 35-week cohort using specific sandwich ELISA kits (eBioscience, Bender MedSystems GmbH, Vienna, Austria), and specific manufacturer’s instructions and previous studies were followed (8;10;13;14). Frozen diaphragm and gastrocnemius muscle specimens were homogenized and protein concentration calculated as described above. Before the start of the assay, samples and reagents were equilibrated to room temperature. In all cases, 15 micrograms of total protein from muscle homogenates were added in a designated well and were immediately incubated with the specific biotin-conjugated antibody for each cytokine on a microplate shaker. Absorbances were read at 450 nm using as a reference filter that of 655 nm in a microplate reader (model 680 Microplate Reader, Bio-Rad Laboratories, Hercules, CA, USA). Intra-assay coefficients of variation for the different cytokines and studies ranged from 4.3% to 8.3%. Inter-assay coefficients of variation for the same cytokines ranged from 8% to 12%. The minimum detectable concentration of each cytokine in muscle homogenates was set to be less than 3.7 and 5.6 pg/mL (TNF-alpha and IL-6, respectively, eBioscience).

**Statistical analysis**

The normality of the study variables was verified using the Shapiro-Wilk test. Physiological and biological results are represented as mean (standard deviation) and the comparisons between all study groups were analyzed using the one-way analysis of variance (ANOVA), in which *Tukey post-hoc* analysis was used to adjust for multiple comparisons among the three study groups in each time-cohort (20 and 35 weeks) independently*.* For the purpose of the investigation, results obtained in the animals and those obtained in the study muscles were subsequently analyzed as follows: 1) non-exposed controls versus lung carcinogenesis mice (U group), 2) non-exposed controls versus lung emphysema-carcinogenesis mice (E-U group), and 3) U versus E-U groups of animals. The sample size chosen was based on previous studies (2;6-8;15-18) and on assumptions of 80% power to detect an improvement of more than 20% in measured outcomes at a level of significance of *P*≤ 0.05. In most biological variables, mean difference between groups was initially estimated at a minimum of 20-25% and standard deviation was approximately 25-30% of the mean value for each of the variables. All statistical analyses were performed using the Statistical Package for the Social Sciences (Portable SPSS, PASW statistics 12.0 version for windows, SPSS Inc., Chicago, IL, USA).

Reference List

(1) Artaechevarria X, Blanco D, de BG, Ceresa M, Perez-Martin D, Bastarrika G, et al. Evaluation of micro-CT for emphysema assessment in mice: comparison with non-radiological techniques. Eur Radiol. 2011 May;21(5):954-62. doi: 10.1007/s00330-010-1982-5 [doi].

(2) Fermoselle C, Sanchez F, Barreiro E. [Reduction of muscle mass mediated by myostatin in an experimental model of pulmonary emphysema]. Arch Bronconeumol. 2011 Dec;47(12):590-8. doi: S0300-2896(11)00300-0 [pii];10.1016/j.arbres.2011.07.008 [doi].

(3) Rudyanto RD, Bastarrika G, de BG, Agorreta J, Montuenga LM, Ortiz-de-Solorzano C, et al. Individual nodule tracking in micro-CT images of a longitudinal lung cancer mouse model. Med Image Anal. 2013 Dec;17(8):1095-105. doi: S1361-8415(13)00109-6 [pii];10.1016/j.media.2013.07.002 [doi].

(4) Marcos JV, Munoz-Barrutia A, Ortiz-de-Solorzano C, Cristobal G. Quantitative Assessment of Emphysema Severity in Histological Lung Analysis. Ann Biomed Eng. 2015 Oct;43(10):2515-29. doi: 10.1007/s10439-015-1251-5 [doi];10.1007/s10439-015-1251-5 [pii].

(5) Munoz-Barrutia A, Ceresa M, Artaechevarria X, Montuenga LM, Ortiz-de-Solorzano C. Quantification of lung damage in an elastase-induced mouse model of emphysema. Int J Biomed Imaging. 2012;2012:734734. doi: 10.1155/2012/734734 [doi]. Pubmed PMID: PMC3503307.

(6) Chacon-Cabrera A, Fermoselle C, Urtreger AJ, Mateu-Jimenez M, Diament MJ, de Kier Joffe ED, et al. Pharmacological strategies in lung cancer-induced cachexia: effects on muscle proteolysis, autophagy, structure, and weakness. J Cell Physiol. 2014 Nov;229(11):1660-72. doi: 10.1002/jcp.24611 [doi].

(7) Chacon-Cabrera A, Fermoselle C, Salmela I, Yelamos J, Barreiro E. MicroRNA expression and protein acetylation pattern in respiratory and limb muscles of Parp-1(-/-) and Parp-2(-/-) mice with lung cancer cachexia. Biochim Biophys Acta. 2015 Dec;1850(12):2530-43. doi: S0304-4165(15)00262-7 [pii];10.1016/j.bbagen.2015.09.020 [doi].

(8) Barreiro E, Puig-Vilanova E, Marin-Corral J, Chacon-Cabrera A, Salazar-Degracia A, Mateu X, et al. Therapeutic Approaches in Mitochondrial Dysfunction, Proteolysis, and Structural Alterations of Diaphragm and Gastrocnemius in Rats With Chronic Heart Failure. J Cell Physiol. 2015 Nov 4; doi: 10.1002/jcp.25241 [doi].

(9) Puig-Vilanova E, Martinez-Llorens J, Ausin P, Roca J, Gea J, Barreiro E. Quadriceps muscle weakness and atrophy are associated with a differential epigenetic profile in advanced COPD. Clin Sci (Lond). 2015 Jun;128(12):905-21. doi: CS20140428 [pii];10.1042/CS20140428 [doi].

(10) Puig-Vilanova E, Rodriguez DA, Lloreta J, Ausin P, Pascual-Guardia S, Broquetas J, et al. Oxidative stress, redox signaling pathways, and autophagy in cachectic muscles of male patients with advanced COPD and lung cancer. Free Radic Biol Med. 2015 Feb;79:91-108. doi: S0891-5849(14)01369-0 [pii];10.1016/j.freeradbiomed.2014.11.006 [doi].

(11) Fermoselle C, Rabinovich R, Ausin P, Puig-Vilanova E, Coronell C, Sanchez F, et al. Does oxidative stress modulate limb muscle atrophy in severe COPD patients? Eur Respir J. 2012 Oct;40(4):851-62. doi: 09031936.00137211 [pii];10.1183/09031936.00137211 [doi].

(12) Barreiro E, Ferrer D, Sanchez F, Minguella J, Marin-Corral J, Martinez-Llorens J, et al. Inflammatory cells and apoptosis in respiratory and limb muscles of patients with COPD. J Appl Physiol. 2011 Sep;111(3):808-17. doi: japplphysiol.01017.2010 [pii];10.1152/japplphysiol.01017.2010 [doi].

(13) Barreiro E, Schols AM, Polkey MI, Galdiz JB, Gosker HR, Swallow EB, et al. Cytokine profile in quadriceps muscles of patients with severe COPD. Thorax. 2008 Feb;63(2):100-7.

(14) Barreiro E, Peinado VI, Galdiz JB, Ferrer E, Marin-Corral J, Sanchez F, et al. Cigarette smoke-induced oxidative stress: A role in chronic obstructive pulmonary disease skeletal muscle dysfunction. Am J Respir Crit Care Med. 2010 Aug 15;182(4):477-88.

(15) Busquets S, Figueras MT, Fuster G, Almendro V, Moore-Carrasco R, Ametller E, et al. Anticachectic effects of formoterol: a drug for potential treatment of muscle wasting. Cancer Res. 2004 Sep 15;64(18):6725-31.

(16) Busquets S, Serpe R, Toledo M, Betancourt A, Marmonti E, Orpi M, et al. L-Carnitine: an adequate supplement for a multi-targeted anti-wasting therapy in cancer. Clin Nutr. 2012 Dec;31(6):889-95. doi: S0261-5614(12)00066-0 [pii];10.1016/j.clnu.2012.03.005 [doi].

(17) Fontes-Oliveira CC, Busquets S, Toledo M, Penna F, Paz AM, Sirisi S, et al. Mitochondrial and sarcoplasmic reticulum abnormalities in cancer cachexia: altered energetic efficiency? Biochim Biophys Acta. 2013 Mar;1830(3):2770-8. doi: S0304-4165(12)00329-7 [pii];10.1016/j.bbagen.2012.11.009 [doi].

(18) Marin-Corral J, Fontes CC, Pascual-Guardia S, Sanchez F, Olivan M, Argiles JM, et al. Redox balance and carbonylated proteins in limb and heart muscles of cachectic rats. Antioxid Redox Signal. 2010 Mar;12(3):365-80.

**LEGENDS**

**Figure S1:** Representative SDS-PAGE stained with Coomassie blue corresponding to the diaphragm (panel A) and gastrocnemius (panel B) muscles of control mice (N=7), U group (N=7) and E-U group (N=8). Abbreviations: E-U, elastase-urethane; PAGE, polyacrylamide gel electrophoresis; SDS, sodium dodecyl sulphate; U, urethane;.

**Figure S2:** Representative immunoblots of GAPDH protein content in the diaphragm (panel A) and gastrocnemius (panel B) muscles of control mice (N=7), U group (N=7) and E-U group (N=8). Abbreviations: a.u., arbitrary units; E-U, elastase-urethane; GAPDH, glyceraldehyde-3-phosphate dehydrogenase; KDa, kilodaltons; MW, molecular weights; OD, optical densities; U, urethane.

**Figure S3:** Immunoblots of skeletal muscle actin protein content in the diaphragm (panel A) and gastrocnemius (panel B) muscles of control mice (N=7), U group (N=7) and E-U group (N=8). Abbreviations: a.u., arbitrary units; E-U, elastase-urethane; KDa, kilodaltons; MW, molecular weights; OD, optical densities; U, urethane.

**Figure S4:** Immunoblots of MyHC protein content in the diaphragm (panel A) and gastrocnemius (panel B) muscles of control mice (diaphragm, N=6; and gastrocnemius, N=7), U group (diaphragm, N=6; and gastrocnemius, N=7) and E-U group (diaphragm, N=6; and gastrocnemius, N=8). Abbreviations: a.u., arbitrary units; E-U, elastase-urethane; KDa, kilodaltons; MW, molecular weights; MyHC, myosin heavy chain; OD, optical densities; U, urethane.

**Figure S5:** Immunoblots of creatine kinase protein content in the diaphragm (panel A) and gastrocnemius (panel B) muscles of control mice (N=7), U group (N=7) and E-U group (N=8). Abbreviations: a.u., arbitrary units; E-U, elastase-urethane; KDa, kilodaltons; MW, molecular weights; OD, optical densities; U, urethane.

**Figure S6:** Immunoblots of carbonic anhydrase-3 protein content in the diaphragm (panel A) and gastrocnemius (panel B) muscles of control mice (N=7), U group (N=7) and E-U group (N=8). Abbreviations: a.u., arbitrary units; E-U, elastase-urethane; KDa, kilodaltons; MW, molecular weights; OD, optical densities; U, urethane.

**Figure S7:** Immunoblots of myogenin protein content in the diaphragm (panel A) and gastrocnemius (panel B) muscles of control mice (N=7), U group (N=7) and E-U group (N=8). Abbreviations: a.u., arbitrary units; E-U, elastase-urethane; KDa, kilodaltons; MW, molecular weights; OD, optical densities; U, urethane.

**Figure S8:** Immunoblots of PPAR-alpha protein content in the diaphragm (panel A) and gastrocnemius (panel B) muscles of control mice (N=7), U group (diaphragm, N=6; and gastrocnemius, N=7) and E-U group (N=8). Abbreviations: a.u., arbitrary units; E-U, elastase-urethane; KDa, kilodaltons; MW, molecular weights; OD, optical densities; PPAR-alpha, peroxisome proliferator-activated receptor-alpha; U, urethane.

**Figure S9:** Immunoblots of PPAR-gamma protein content in the diaphragm (panel A) and gastrocnemius (panel B) muscles of control mice (N=7), U group (diaphragm, N=6; and gastrocnemius, N=7) and E-U group (N=8). Abbreviations: a.u., arbitrary units; E-U, elastase-urethane; KDa, kilodaltons; MW, molecular weights; OD, optical densities; PPAR-gamma, peroxisome proliferator-activated receptor-gamma; U, urethane.

**Figure S10:** Immunoblots of PGC-1 alpha protein content in the diaphragm (panel A) and gastrocnemius (panel B) muscles of control mice (N=7), U group (diaphragm, N=6; and gastrocnemius, N=7) and E-U group (N=8). Abbreviations: a.u., arbitrary units; E-U, elastase-urethane; KDa, kilodaltons; MW, molecular weights; OD, optical densities; PGC-1, peroxisome proliferator-activated receptor gamma coactivator-1alpha; U, urethane.

**Figure S11:** Immunoblots of MDA-protein adducts in the diaphragm (panel A) and gastrocnemius (panel B) muscles of control mice (N=7), U group (N=7) and E-U group (N=8). Abbreviations: a.u., arbitrary units; E-U, elastase-urethane; KDa, kilodaltons; MDA, malondialdehyde; MW, molecular weights; OD, optical densities; U, urethane.

**Figure S12:** Immunoblots of SOD1 protein content in the diaphragm (panel A) and gastrocnemius (panel B) muscles of control mice (N=7), U group (N=7) and E-U group (N=8). Abbreviations: a.u., arbitrary units; E-U, elastase-urethane; KDa, kilodaltons; MW, molecular weights; OD, optical densities; SOD1, superoxide dismutase isoform 1; U, urethane.

**Figure S13:** Immunoblots of SOD2 protein content in the diaphragm (panel A) and gastrocnemius (panel B) muscles of control mice (diaphragm, N=6; and gastrocnemius, N=7), U group (diaphragm, N=5; and gastrocnemius, N=7) and E-U group (diaphragm, N=7; and gastrocnemius, N=8). Abbreviations: a.u., arbitrary units; E-U, elastase-urethane; KDa, kilodaltons; MW, molecular weights; OD, optical densities; SOD2, superoxide dismutase isoform 2; U, urethane.

**Figure S14:** Immunoblots of catalase protein content in the diaphragm (panel A) and gastrocnemius (panel B) muscles of control mice (N=7), U group (N=7) and E-U group (N=8). Abbreviations: a.u., arbitrary units; E-U, elastase-urethane; KDa, kilodaltons; MW, molecular weights; OD, optical densities; U, urethane.

**Figure S15:** Immunoblots of total protein ubiquitination in the diaphragm (panel A) and gastrocnemius (panel B) muscles of control mice (N=7), U group (N=7) and E-U group (N=8). Abbreviations: a.u., arbitrary units; E-U, elastase-urethane; KDa, kilodaltons; MW, molecular weights; OD, optical densities; U, urethane.

**Figure S16:** Immunoblots of E2_14k_ protein content in the diaphragm (panel A) and gastrocnemius (panel B) muscles of control mice (N=7), U group (N=7) and E-U group (N=8). Abbreviations: a.u., arbitrary units; E-U, elastase-urethane; E2_14k_, ubiquitin-conjugating enzyme E2 (14k); KDa, kilodaltons; MW, molecular weights; OD, optical densities; U, urethane.

**Figure S17:** Immunoblots of MURF-1 protein content in the diaphragm (panel A) and gastrocnemius (panel B) muscles of control mice (N=7), U group (N=7) and E-U group (N=8). Abbreviations: a.u., arbitrary units; E-U, elastase-urethane; KDa, kilodaltons; MW, molecular weights; MURF-1, muscle ring finger protein 1;OD, optical densities; U, urethane.

**Figure S18:** Immunoblots of calpain-1 protein content in the diaphragm (panel A) and gastrocnemius (panel B) muscles of control mice (N=7), U group (N=7) and E-U group (N=8). Abbreviations: a.u., arbitrary units; E-U, elastase-urethane; KDa, kilodaltons; MW, molecular weights; OD, optical densities; U, urethane.

**Figure S19:** Immunoblots of p62 protein content in the diaphragm (panel A) and gastrocnemius (panel B) muscles of control mice (diaphragm, N=8; and gastrocnemius, N=7), U group (diaphragm, N=6; and gastrocnemius, N=7) and E-U group (N=8). Abbreviations: a.u., arbitrary units; E-U, elastase-urethane; KDa, kilodaltons; MW, molecular weights; OD, optical densities; p62, nucleoporin p62; U, urethane.

**Figure S20:** Immunoblots of beclin-1 protein content in the diaphragm (panel A) and gastrocnemius (panel B) muscles of control mice (N=7), U group (N=7) and E-U group (N=8). Abbreviations: a.u., arbitrary units; E-U, elastase-urethane; KDa, kilodaltons; MW, molecular weights; OD, optical densities; U, urethane.

**Figure S21:** Immunoblots of LC3 II/ LC3 I levels in the diaphragm (panel A) and gastrocnemius (panel B) muscles of control mice (N=7), U group (N=7) and E-U group (N=8). Abbreviations: a.u., arbitrary units; E-U, elastase-urethane; KDa, kilodaltons; LC3, light chain 3; MW, molecular weights; OD, optical densities; U, urethane.


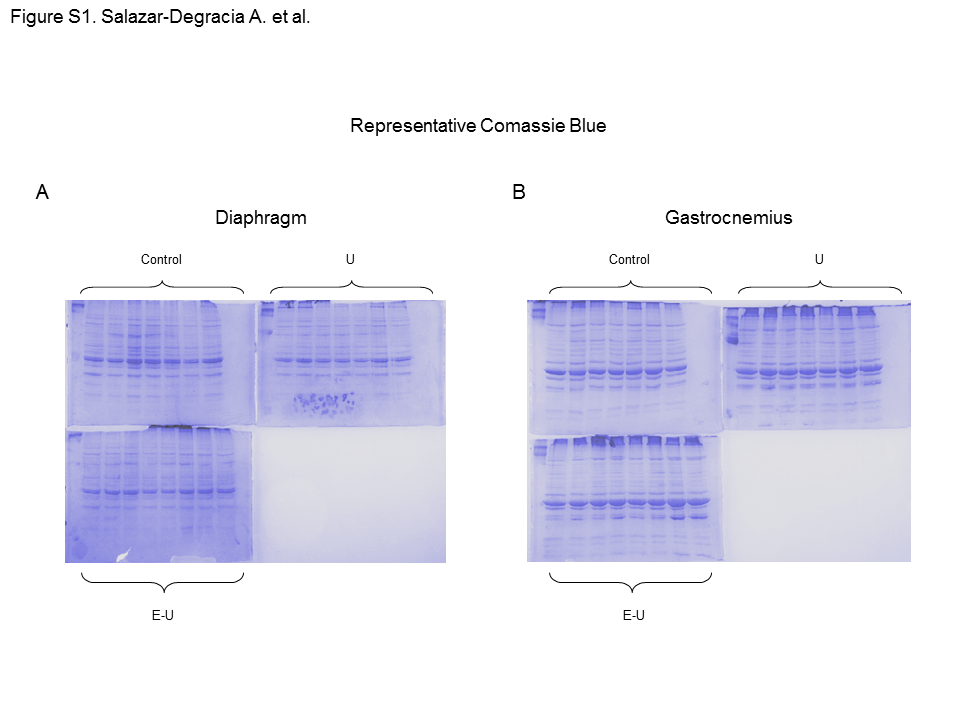


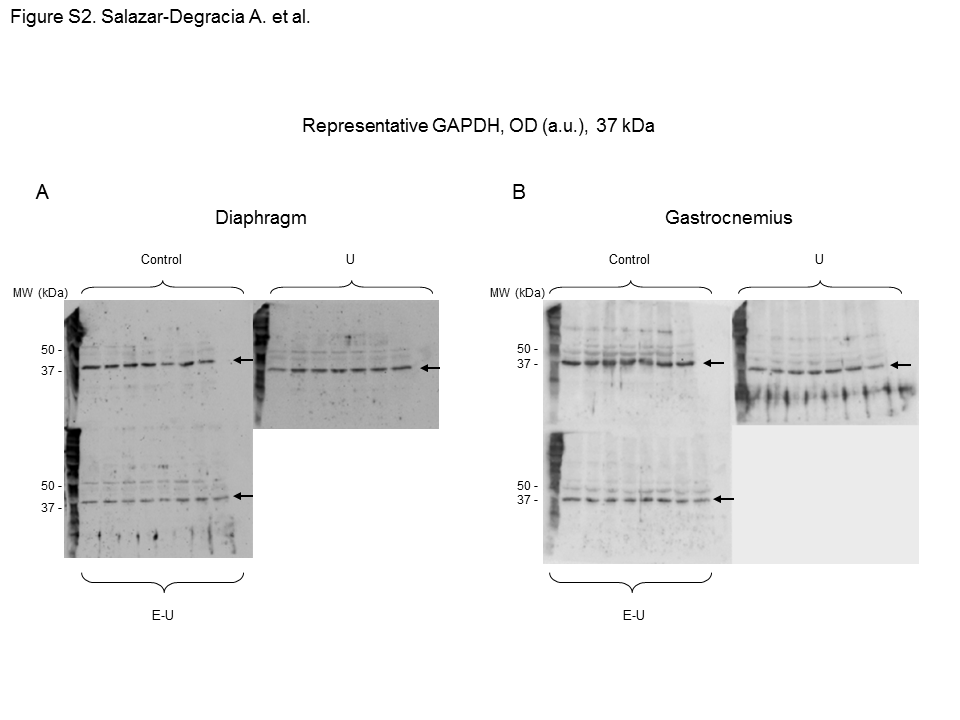


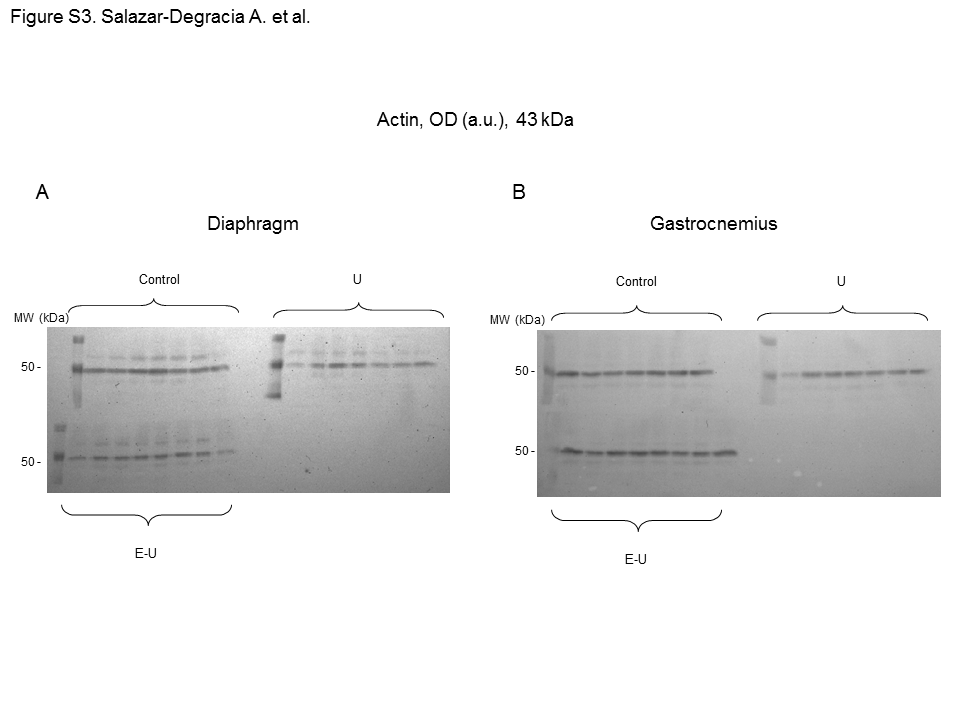


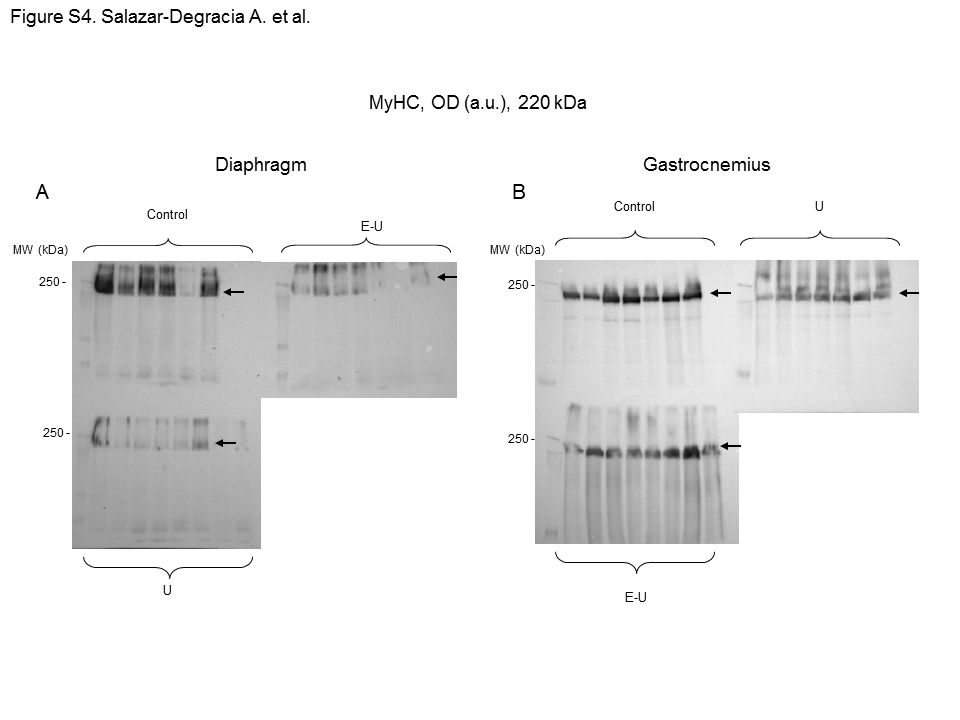


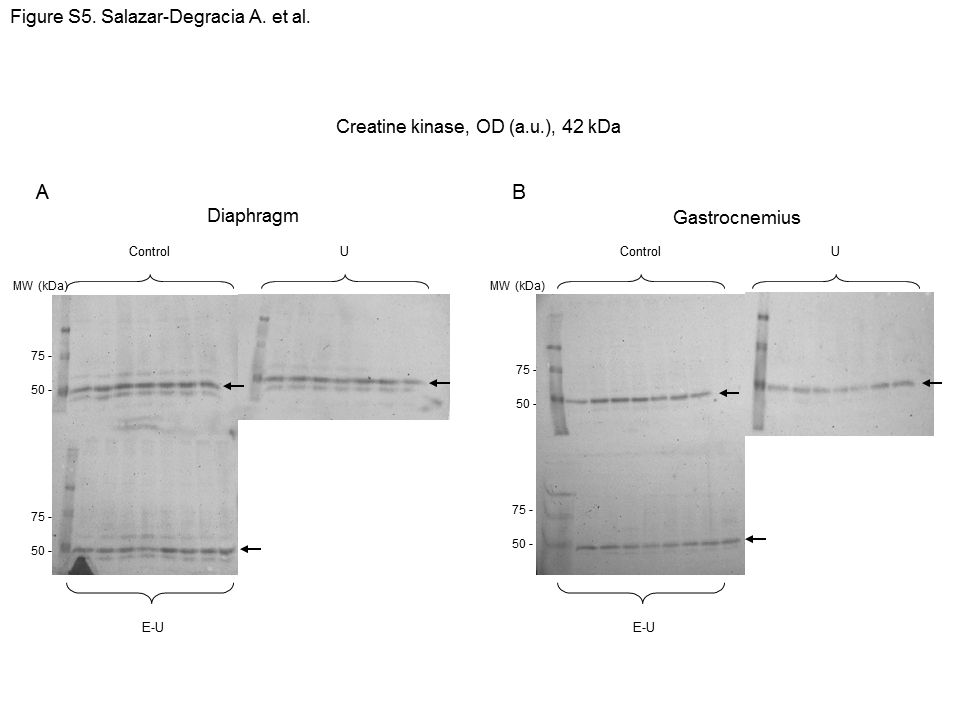


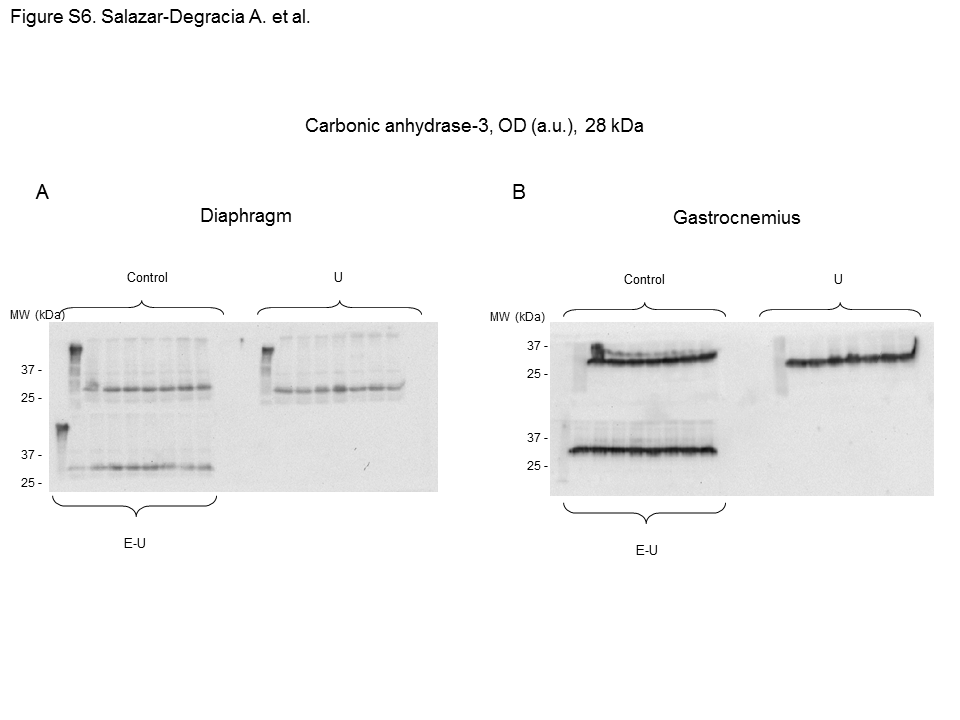


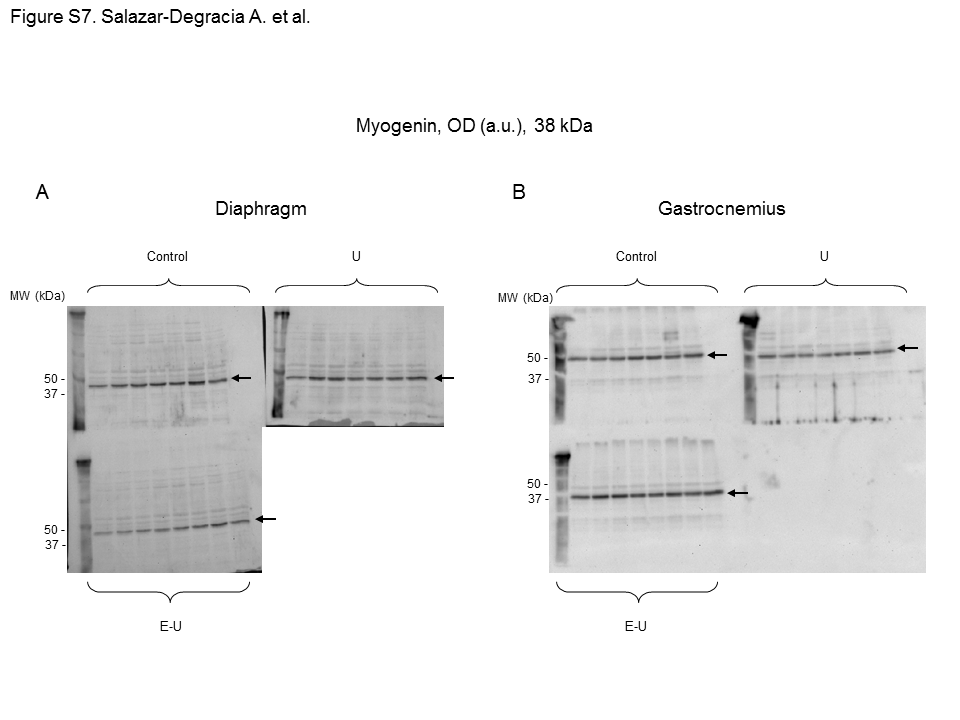


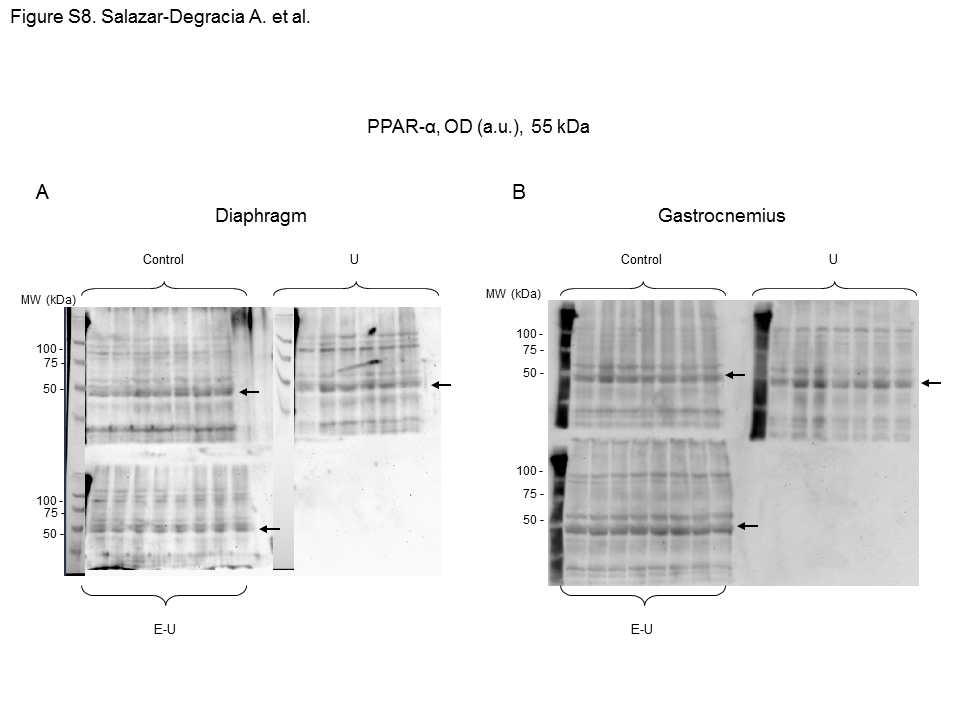


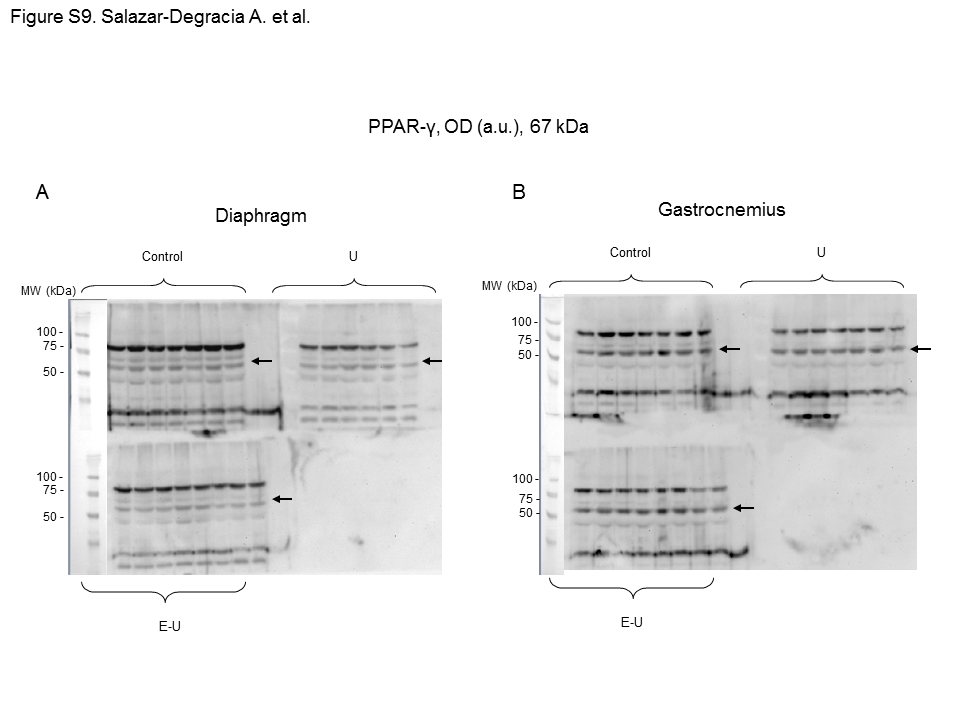


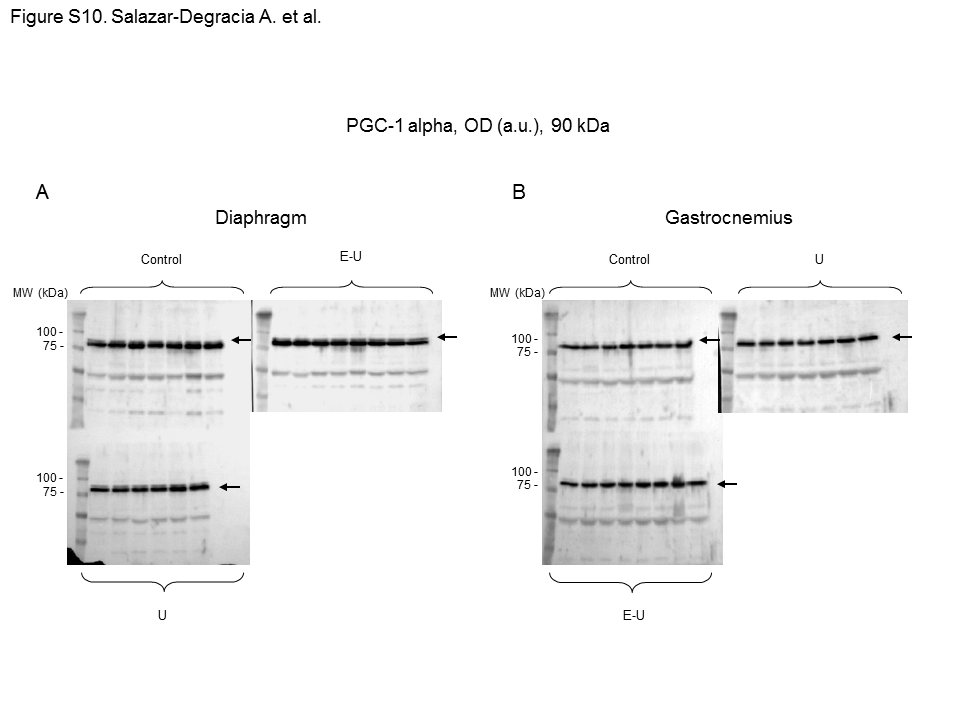


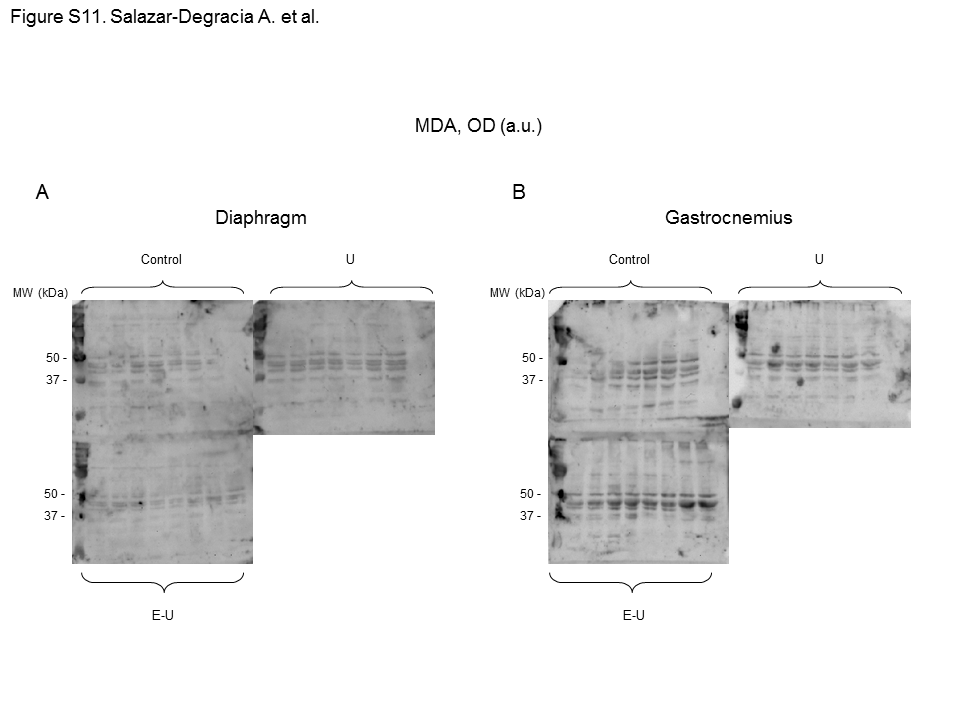


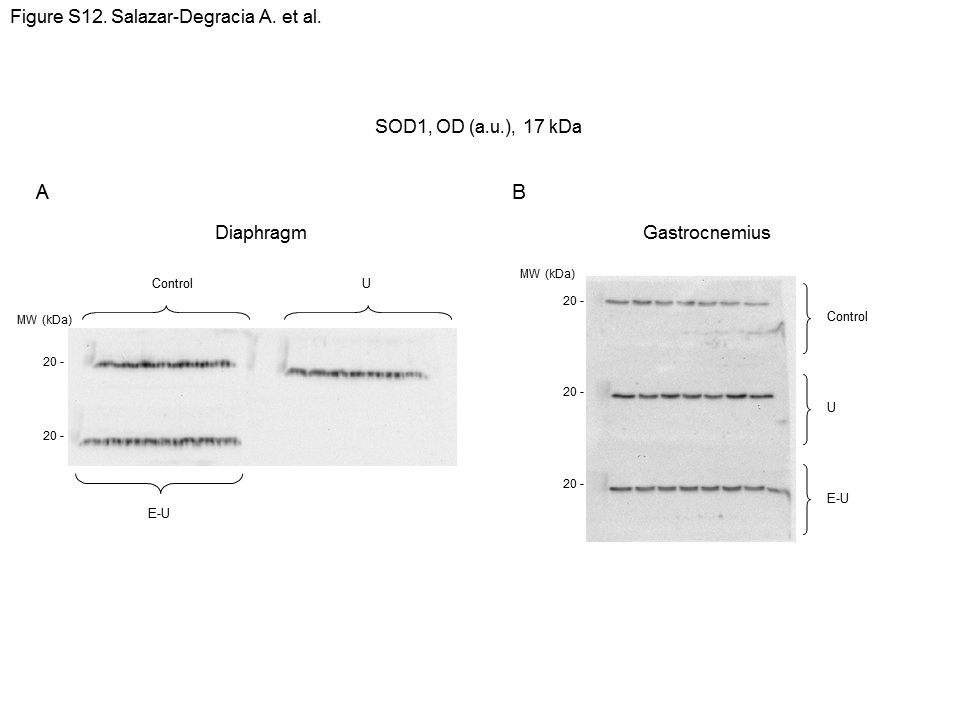


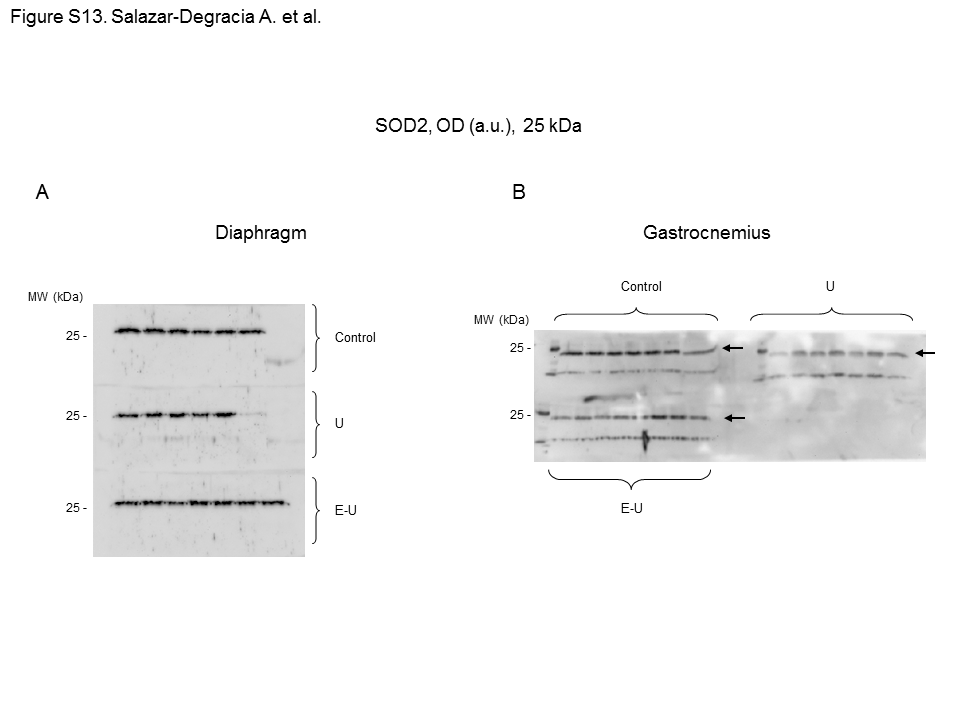


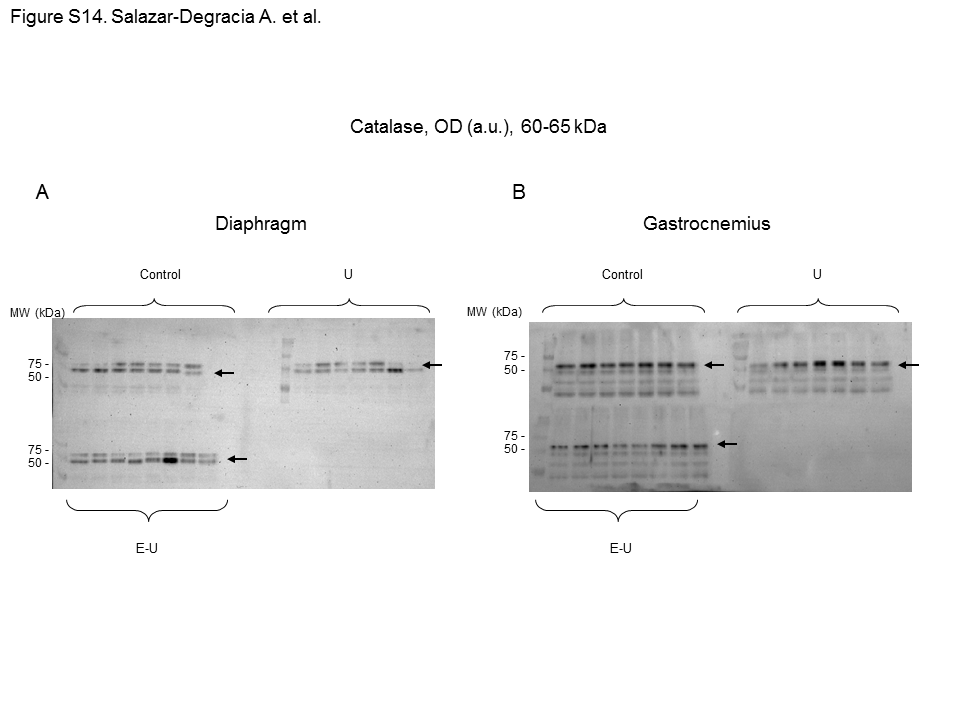


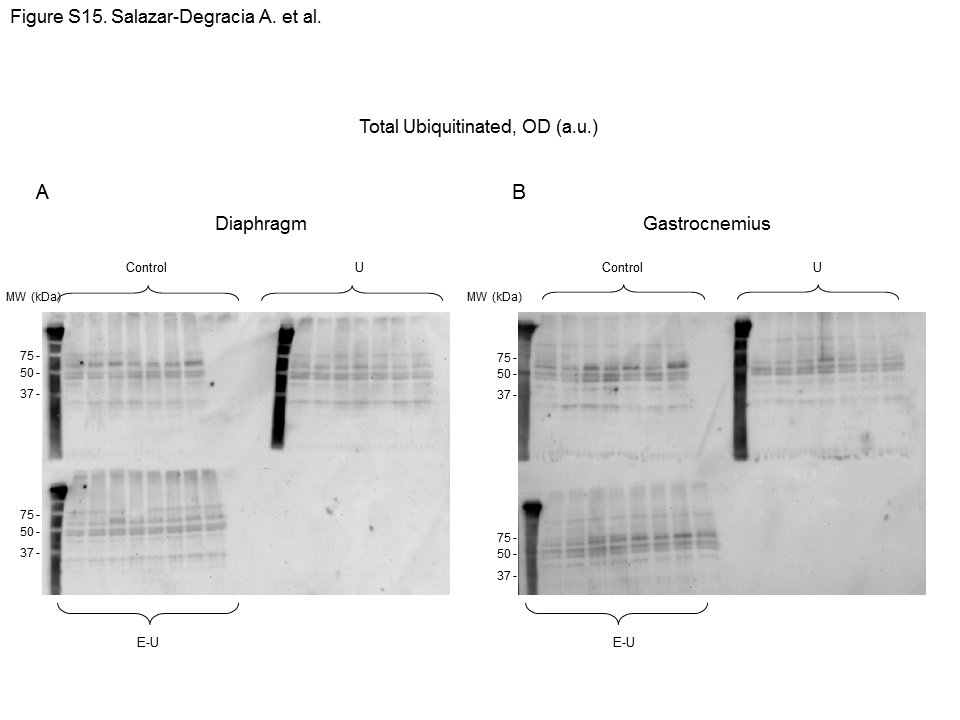


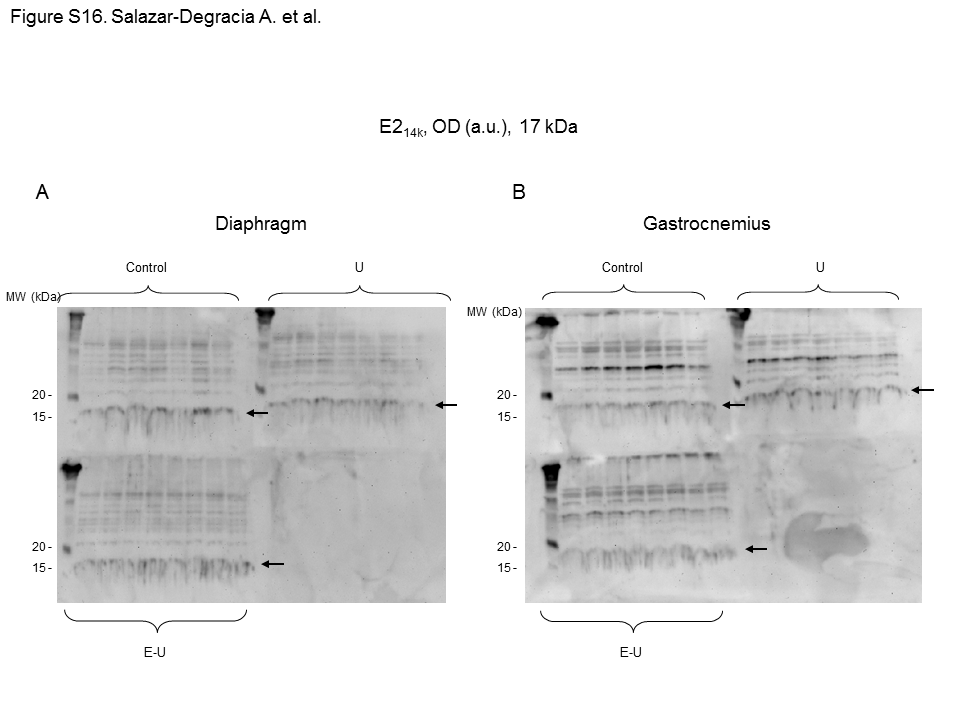


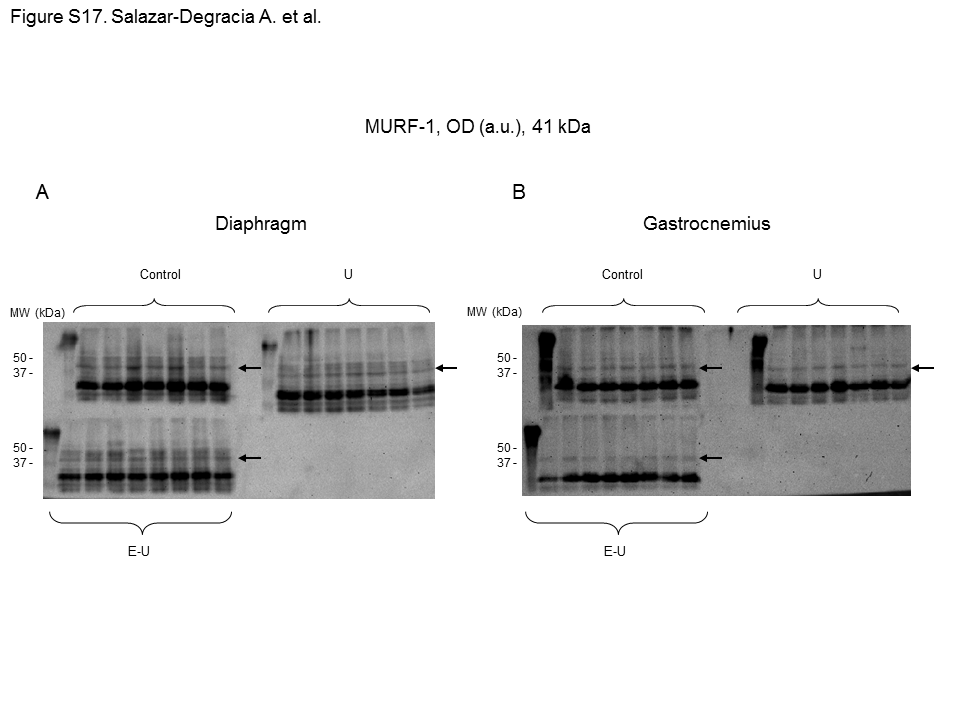


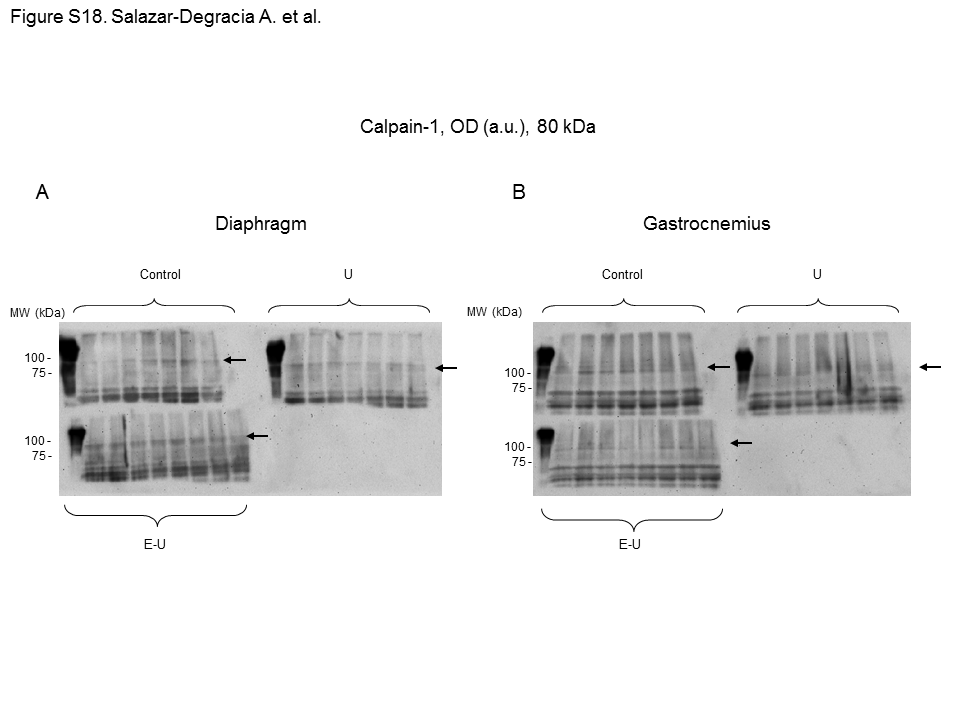


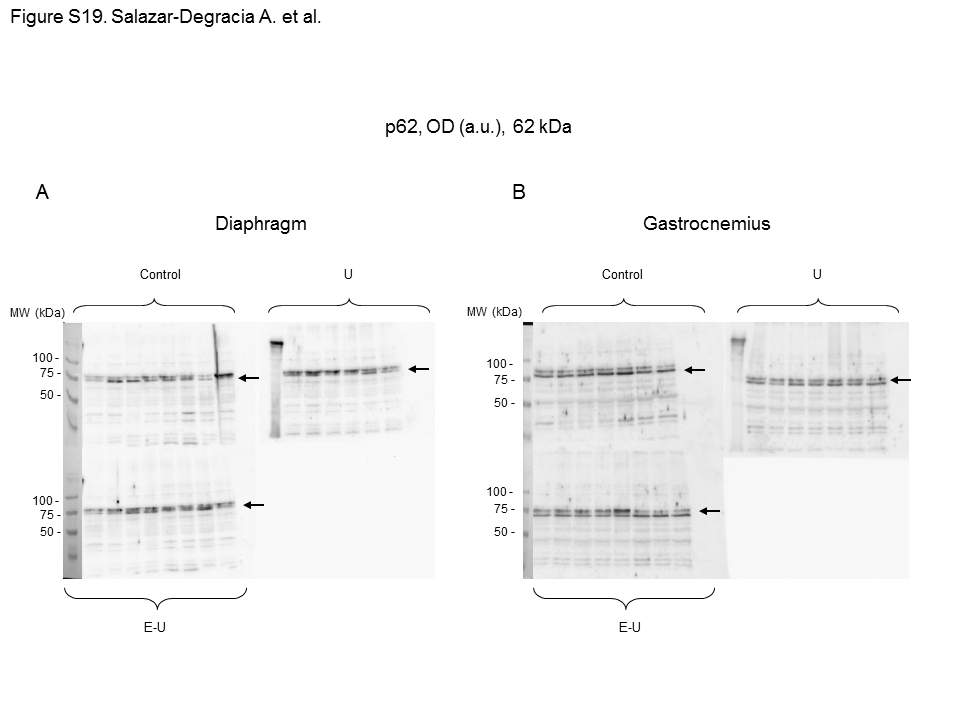


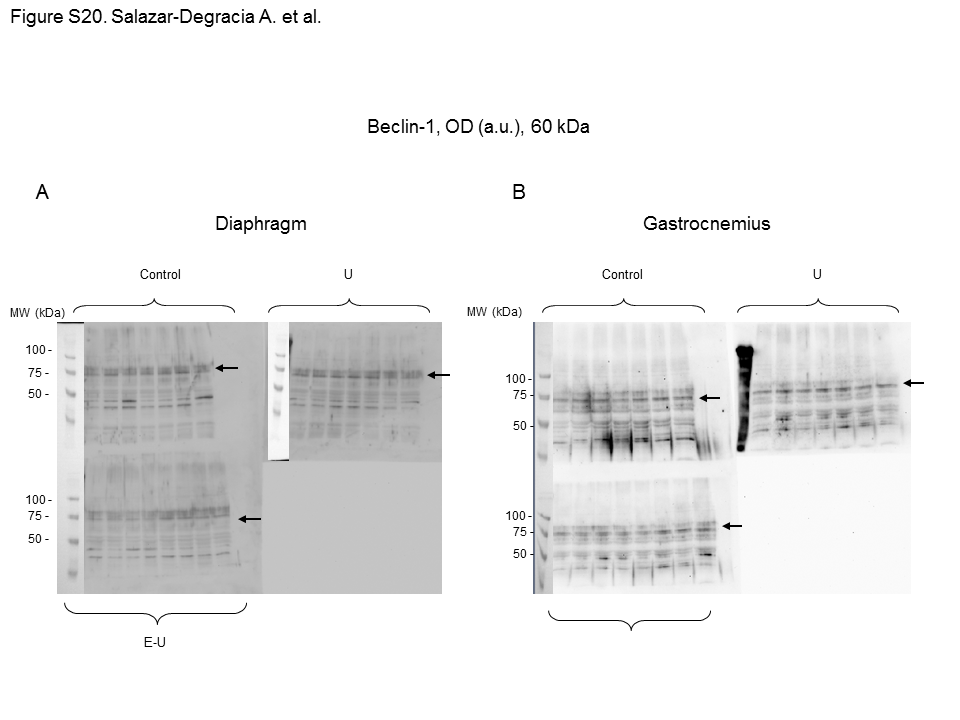


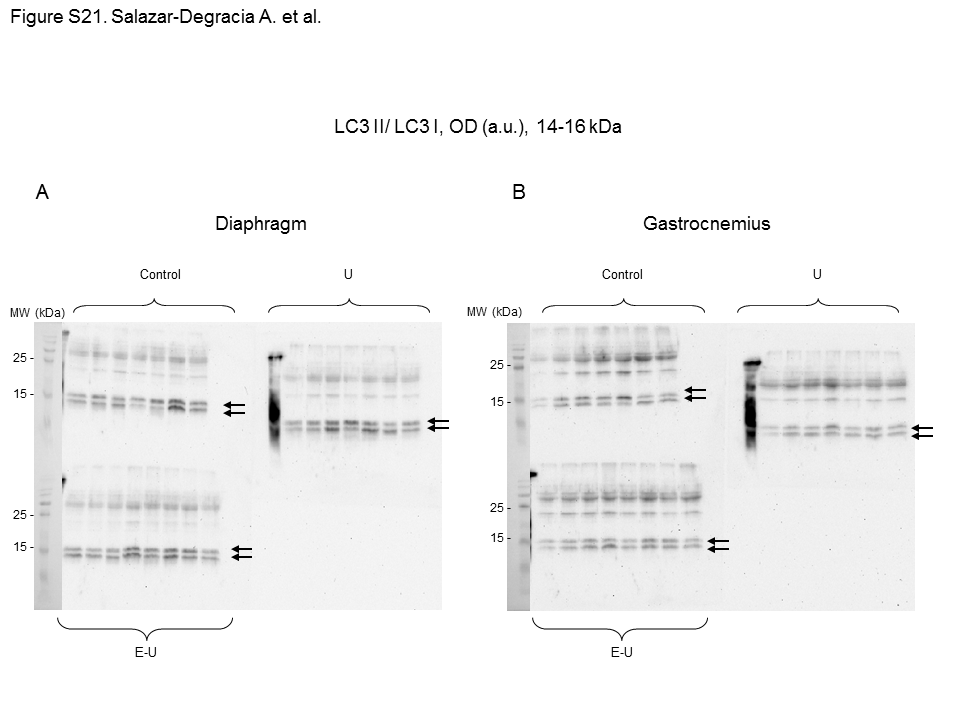

Supplement: Supplementary file 1 — 10.1186/s12967-016-1003-9 Online data supplement. [file 12967_2016_1003_MOESM1_ESM.doc]
